# Pan-Genome-Wide Analysis of *Pantoea ananatis* Identified Genes Linked to Pathogenicity in Onion

**DOI:** 10.3389/fmicb.2021.684756

**Published:** 2021-08-19

**Authors:** Gaurav Agarwal, Divya Choudhary, Shaun P. Stice, Brendon K. Myers, Ronald D. Gitaitis, Stephanus N. Venter, Brian H. Kvitko, Bhabesh Dutta

**Affiliations:** ^1^Department of Plant Pathology, Coastal Plain Experimental Station, University of Georgia, Tifton, GA, United States; ^2^Department of Plant Pathology, University of Georgia, Athens, GA, United States; ^3^Department of Biochemistry, Genetics and Microbiology, Forestry and Agricultural Biotechnology Institute, University of Pretoria, Pretoria, South Africa

**Keywords:** pan-genome, horizontal gene transfer, genome-wide association study, SNPs, presence and absence variants

## Abstract

Pantoea ananatis, a gram negative and facultative anaerobic bacterium is a member of a *Pantoea* spp. complex that causes center rot of onion, which significantly affects onion yield and quality. This pathogen does not have typical virulence factors like type II or type III secretion systems but appears to require a biosynthetic gene-cluster, HiVir/PASVIL (located chromosomally comprised of 14 genes), for a phosphonate secondary metabolite, and the ‘*alt*’ gene cluster (located in plasmid and comprised of 11 genes) that aids in bacterial colonization in onion bulbs by imparting tolerance to thiosulfinates. We conducted a deep pan-genome-wide association study (pan-GWAS) to predict additional genes associated with pathogenicity in *P. ananatis* using a panel of diverse strains (*n* = 81). We utilized a red-onion scale necrosis assay as an indicator of pathogenicity. Based on this assay, we differentiated pathogenic (*n* = 51)- vs. non-pathogenic (*n* = 30)-strains phenotypically. Pan-genome analysis revealed a large core genome of 3,153 genes and a flexible accessory genome. Pan-GWAS using the presence and absence variants (PAVs) predicted 42 genes, including 14 from the previously identified HiVir/PASVIL cluster associated with pathogenicity, and 28 novel genes that were not previously associated with pathogenicity in onion. Of the 28 novel genes identified, eight have annotated functions of site-specific tyrosine kinase, N-acetylmuramoyl-L-alanine amidase, conjugal transfer, and HTH-type transcriptional regulator. The remaining 20 genes are currently hypothetical. Further, a core-genome SNPs-based phylogeny and horizontal gene transfer (HGT) studies were also conducted to assess the extent of lateral gene transfer among diverse *P. ananatis* strains. Phylogenetic analysis based on PAVs and whole genome multi locus sequence typing (wgMLST) rather than core-genome SNPs distinguished red-scale necrosis inducing (pathogenic) strains from non-scale necrosis inducing (non-pathogenic) strains of *P. ananatis*. A total of 1182 HGT events including the HiVir/PASVIL and *alt* cluster genes were identified. These events could be regarded as a major contributing factor to the diversification, niche-adaptation and potential acquisition of pathogenicity/virulence genes in *P. ananatis*.

## Introduction

The genus *Pantoea* currently has 27 recognized species; five of which are known to cause disease-associated losses in several crops (Arnold et al., 2003; Cruz et al., 2007; Coutinho and Venter, 2009; Bankevich et al., 2012; De Maayer et al., 2014). Three species of *Pantoea*, namely, *P. ananatis, P. agglomerans* and *P. stewartii* subsp. *indologenes* are responsible for more than 80% of the reported cases of disease in onions (Kini et al., 2019). *Pantoea ananatis*, a Gram-negative and facultative anaerobic bacterium that belongs to the Erwiniaceae (previously assigned to enterobacteriaceae) is part of a *Pantoea* spp. complex (also including *P. allii, P. agglomerans*, and *P. stewartii* subs. *indologenes*), which causes center rot of onion (Gitaitis et al., 2002; Walcott et al., 2002; Stumpf et al., 2018). Foliar symptoms primarily appear as white streaks and water-soaked lesions, and more advanced infections result in complete collapse of foliar tissues, discoloration and softening of specific scale layers in the bulb. Under favorable conditions, center rot can result in 100% losses in the field. In Georgia, late maturing varieties are more susceptible to center rot than early maturing varieties (Gitaitis and Gay, 1997; Carr et al., 2013; Agarwal et al., 2019). Out of the four species in the *Pantoea* spp. complex, *P. ananatis* has been associated predominantly with center rot, in Georgia (Dutta et al., 2014). *Pantoea ananatis* has been identified in other onion-growing regions of the United States, including Colorado (Schwartz and Otto, 2000), Michigan (Schwartz and Mohan, 2008), New York (Carr et al., 2010), and Pennsylvania (Pfeufer et al., 2015). The bacterium can be found as an epiphyte on crop and weed plants (Gitaitis et al., 2002) or as an endophyte in maize kernels and rice seeds (Okunishi et al., 2005; Rijavec et al., 2007). Apart from its epiphytic and endophytic niche in crops and weeds, *P. ananatis* can be disseminated through infected onion seed and contaminated insect vectors (thrips) to onion crops (Gitaitis et al., 2003; Dutta et al., 2014).

*Pantoea ananatis*, unlike many other phytopathogenic bacteria, lacks genes that code for type II, III and IV protein secretory systems that are associated with pathogenicity and virulence (De Maayer et al., 2014). Recent studies utilized whole genome/small RNA sequencing that aided in identifying some virulence factors associated with *P. ananatis* in onion. These virulence factors are flagellar and pilin motility factors (Weller-Stuart et al., 2017) and a global virulence regulator (Hfq, an RNA chaperone) associated with quorum sensing and biofilm production (Shin et al., 2019). However, these genetic factors are present in both onion-pathogenic and non-pathogenic strains. Previous comparative genomics studies on *P. ananatis* were carried out to identify pathogenicity-related regions in the genome using a small sub-set of strains (2 to 10 strains) (De Maayer et al., 2014; Sheibani-Tezerji et al., 2015; Asselin et al., 2018; Stice et al., 2018). Asselin et al. (2018) proposed a biosynthetic gene-cluster, HiVir/PASVIL (High Virulence also known as PASVIL; *Pantoea ananatis* specific virulence locus) that encodes a proposed phosphonate or phosphonate secondary metabolite cluster located on the chromosome, has been demonstrated to be associated with onion foliar and bulb necrosis (Asselin et al., 2018; Takikawa and Kubota, 2018). Recently, Polidore et al. (2021) identified this gene cluster to encode for at least three phosphonates two of which are characterized and named as pantaphos 2-(hydroxy[phosphono]methyl) maleate and 2- (phosphonomethyl) maleate.

In addition, Stice et al. (2018) showed that a megaplasmid-borne onion virulence region (OVR) in *P. ananatis* is correlated with onion virulence. In a recent study (Stice et al., 2020) showed that the OVRA cluster contains 11 genes that are critical for colonizing necrotized bulb tissue. This gene cluster was described as the ‘*alt’* cluster that imparts bacterial tolerance to the thiosulfinate ‘allicin’ in onion bulbs. Presence or absence of genes in these gene clusters (14 genes in HiVir/PASVIL and 11 genes in ‘*alt*’) may dictate the pathogenic potential of *P. ananatis* in onion. However, a large-scale genomic study utilizing diverse *P. ananatis* strains with pathogenic or non-pathogenic phenotype has not been done to evaluate this aspect.

The pan-genome is defined as the total number of non-redundant genes present in a given clade, amounting to a given clade’s entire genomic repertoire, and encodes for all possible ecological niches of the strains examined (Tettelin et al., 2005; Snipen et al., 2009). A pan-genome typically contains core genes, accessory genes and the strain-specific genes. Defining the soft-core genes helps in obtaining a robust estimate of the core genes. The core and soft-core clusters together represented a pool of highly conserved genes, which may provide insights into the evolutionary history of a bacterial pathogen. The cloud cluster include genes that are strain specific or unique in the pan-genome. Both the shell and cloud clusters represent a subset of flexible (accessory) genome that reflects life-style and adaptation of bacterial strains to the environments in which they reside (Nelson and Stegen, 2015). Another significance of a pan-genome is that it can provide a greater resolution and aids in reconstructing bacterial phylogeny in a more reliable way than single or multiple gene-based phylogeny. The pan-genome provides an overview of the entire gene set (100% of the genomes) of a given population, unlike a 16S rRNA phylogeny that represents only a tiny fraction of the genome (∼0.07%), or multi-locus sequence analysis (MLSA) involving house-keeping genes (∼0.2%). De Maayer et al. (2014) analyzed the pan-genomes of eight sequenced *P. ananatis* strains isolated from different sources and identified factors that can potentially explain their ecological niche and their interactions with the environment and host. Asselin et al. (2018) and Stice et al. (2018, 2020) utilized comparative genomic approaches to identify HiVir/PASVIL and the *alt* gene clusters, respectively in onion-pathogenic *P. ananatis*. Further pan-genomic studies with large strain collection from diverse isolation sources and onion-pathogenic potential may shed further light on the novel pathogenic factors responsible for center rot disease in onion. In addition, the openness of a bacterial pan-genome reflects the diversity of the gene pool among the strains of the same bacterial species. The addition of new genomes to an existing pan-genome can significantly alter the size of an open pan-genome in contrast to a closed pan-genome (Vernikos et al., 2015). This aspect was evaluated earlier in *P. ananatis* using a small sub-set of pan-genomes (*n* = 8), which needs further evaluation using a large and diverse set of *P. ananatis* strains (De Maayer et al., 2014).

Bacterial genomes are labile entities, fluctuating both in size and gene content through time. Such genome fluctuations are maintained by the counteracting processes of gene gain and loss (Touchon et al., 2009; Nowell et al., 2014). Horizontal gene transfer (HGT) can result in the replacement of genetic segments with donor homologs, often within species via homologous recombination, or via. acquisition of new genetic material. The gene-presence-absence variants (PAVs) are primarily the result of various HGT events in bacterial evolution. Such HGT events have not been fully explored in *P. ananatis* particularly in onion-pathosystem. In this manuscript, we therefore explored HGT events among *P. ananatis* strains isolated from diverse sources including onion, weeds and thrips with or without pathogenic potential on onion.

Genomic variants identified using pan-genomic analysis can be associated with the unique phenotypic characteristics of an organism using computational methods to identify the genetic basis of phenotypic variations. Such computational association methods have been utilized in research of humans (Visscher et al., 2017; Chen et al., 2018; Gong et al., 2019), domesticated plants (Varshney et al., 2017; Varshney et al., 2019), animals (Bolormaa et al., 2011), and bacteria (Chen and Shapiro, 2015; Falush, 2016; Epstein et al., 2018). This approach is widely used as a genome wide association study (GWAS) that associates genomic characteristics such as single nucleotide polymorphisms (SNPs), insertion and deletions (InDels) or copy number variants (CNVs) or PAVs with the phenotypic characteristics of an organism. This approach has not been used in *P. ananatis* and may aid in identifying novel factors responsible for pathogenicity in onion. In this manuscript, we describe the use of a pan-GWAS approach that predicted novel genes associated with onion-pathogenicity in *P. ananatis* and provide genomic evidence that presence of entire HiVir/PASVIL and ‘*alt*’ clusters do not warrant an onion pathogenic phenotypic.

## Materials and Methods

### Bacterial Strains, Identification, and Culture Preparation

Eighty-one *P. ananatis* strains used in this study were isolated from onion foliage, bulb and seeds as well as from weeds and thrips throughout the state of Georgia (Table 1). These strains were stored in a 15% aqueous glycerol solution at -80°C. The source, year of isolation, and county of origin in Georgia for these strains are listed (Table 1). Strains were identified as *P. ananatis* by their colony morphology and physiological characteristics as Gram-negative, facultatively anaerobic, positive for indole production, negative for nitrate reductase, and phenylalanine deaminase and using *P. ananatis*-specific primers (Walcott et al., 2002). Strains that were isolated from onion plants were designated as “PNA” and strains from non-onion sources (e.g., weeds or thrips) were identified as “PANS.”

**TABLE 1:** List of *Pantoea ananatis* strains used in this study along with their pan-genomes details, geographical locations, pathogenic phenotypes and isolation sources.

Strains	BioSample ID	Genome accession	Size (Mb)	Contigs (>300 bp)	CDSs	Genes/mRNAs	GC (%)	Location	Red-onion scale necrosis (±)*	Source
PNA_99_9	SAMN14604903	JABEBS000000000	5.13	151	4696	4766	53.32	Tattnall Co.	+	Onion
PNA_99_8	SAMN14604904	JABEBR000000000	4.99	56	4549	4617	53.41	Wheeler Co.	+	Onion
PNA_99_7	SAMN14604905	JABEBQ000000000	5.05	36	4662	4728	53.42	Tattnall Co.	+	Onion
PNA_99_6	SAMN14604906	JABEBP000000000	5.1	43	4710	4778	53.36	Toombs Co.	+	Onion
PNA_99_3	SAMN14604907	JABEBO000000000	5.14	39	4659	4726	53.29	Tift Co.	+	Onion
PNA_99_2	SAMN14604908	JABEBN000000000	4.98	38	4540	4607	53.49	Tattnall Co.	+	Onion
PNA_99_14	SAMN14604909	JABEBM000000000	4.96	67	4549	4625	53.44	Toombs Co.	+	Onion
PNA_99_1	SAMN14604910	JABEBL000000000	4.91	67	4489	4556	53.48	MT Vernon	+	Onion
PNA_98_8	SAMN14604911	JABEBK000000000	4.93	31	4547	4618	53.45	Vidalia Region	+	Onion
PNA_98_7	SAMN14604912	JABEBJ000000000	5.08	43	4678	4748	53.64	Tift Co.	−	Onion
PNA_98_3	SAMN14604913	JABEBI000000000	5.47	413	4997	5067	53.18	Dougherty	−	Onion
PNA_98_2	SAMN14604914	JABEBH000000000	5.14	38	4693	4757	53.3	Tift Co.	+	Onion
PNA_98_12	SAMN14604915	JABEBG000000000	5.73	942	4990	5061	53.43	Toombs Co.	+	Onion
PNA_98_11	SAMN14604916	JABEBF000000000	5.8	954	5065	5136	53.59	Evans Co.	−	Onion
PNA_98_1	SAMN14604917	JABEBE000000000	5.67	796	4938	5016	53.47	Tattnall Co.	+	Onion
PNA_97_3	SAMN14604918	JABEBD000000000	4.96	36	4545	4613	53.25	Toombs Co.	+	Onion
PNA_97_11	SAMN14604919	JABEBC000000000	5.09	38	4695	4762	53.4	Toombs Co.	+	Onion
PNA_92_7	SAMN14604920	JABEBB000000000	5.31	373	4714	4780	53.44	Vidalia Region	+	Onion
PNA_200_8	SAMN14604921	JABEBA000000000	5	36	4606	4680	53.52	Tift Co.	−	Onion
PNA_200_7	SAMN14604922	JABEAZ000000000	5.01	70	4567	4637	53.27	Tift Co.	+	Onion
PNA_200_3	SAMN14604923	JABEAY000000000	5.01	39	4610	4686	53.52	Tift Co.	−	Onion
PNA_200_12	SAMN14604924	JABEAX000000000	4.92	34	4517	4591	53.48	Tift Co.	+	Onion
PNA_200_11	SAMN14604925	JABEAW000000000	4.96	32	4545	4616	53.49	Tift Co.	+	Onion
PNA_200_10	SAMN14604926	JABEAV000000000	5.01	25	4589	4655	53.48	Tift Co.	+	Onion
PNA_18_9s	SAMN14604927	JABEAU000000000	5.52	342	5110	5178	52.94	Vidalia Region	+	Onion
PNA_18_8s	SAMN14604928	JABEAT000000000	5.01	34	4651	4714	53.42	Vidalia Region	−	Onion
PNA_18_7s	SAMN14604929	JABEAS000000000	4.88	52	4422	4492	53.49	Vidalia Region	+	Onion
PNA_18_6s	SAMN14604930	JABEAR000000000	5.02	46	4648	4719	53.42	Vidalia Region	−	Onion
PNA_18_5s	SAMN14604931	JABEAQ000000000	5.61	1027	5054	5182	53.03	Vidalia Region	+	Onion
PNA_18_5	SAMN14604932	JABEAP000000000	4.86	46	4429	4499	53.49	Vidalia Region	+	Onion
PNA_18_3s	SAMN14604933	JABEAO000000000	4.95	34	4524	4591	53.64	Vidalia Region	+	Onion
PNA_18_2	SAMN14604934	JABEAN000000000	4.95	26	4526	4591	53.25	Vidalia Region	+	Onion
PNA_18_10s	SAMN14604935	JABEAM000000000	5.08	80	4668	4738	53.42	Vidalia Region	−	Onion
PNA_18_10	SAMN14604936	JABEAL000000000	5.53	433	4932	5006	53.56	Vidalia Region	−	Onion
PNA_18_1	SAMN14604937	JABEAK000000000	4.96	27	4530	4603	53.25	Vidalia Region	+	Onion
PNA_15_3	SAMN14604938	JABEAJ000000000	4.93	33	4544	4619	53.24	Tattnall Co.	−	Onion
PNA_15_1	SAMN14604939	JABEAI000000000	4.99	31	4588	4662	53.34	Tattnall Co.	+	Onion
PNA_14_2	SAMN14604940	JABEAH000000000	4.98	46	4615	4691	53.35	Lyons	−	Onion
PNA_13_1	SAMN14604941	JABEAG000000000	4.87	46	4498	4564	53.39	Lyons	−	Onion
PNA_11_1	SAMN14604942	JABEAF000000000	4.91	30	4476	4549	53.41	Vidalia Region	−	Onion
PNA_08_1	SAMN14604943	JABEAE000000000	4.88	31	4486	4561	53.59	Tattnall Co.	+	Onion
PNA_07_7	SAMN14604944	JABEAD000000000	4.9	26	4495	4568	53.43	Toombs Co.	+	Onion
PNA_07_5	SAMN14604945	JABEAC000000000	4.94	27	4551	4621	53.6	Wayne Co.	+	Onion
PNA_07_22	SAMN14604946	JABEAB000000000	4.91	62	4534	4598	53.6	Tift Co.	−	Onion
PNA_07_14	SAMN14604947	JABEAA000000000	4.93	32	4503	4571	53.52	Toombs Co.	−	Onion
PNA_07_13	SAMN14604948	JABDZZ000000000	4.93	29	4527	4594	53.48	Toombs Co.	−	Onion
PNA_07_10	SAMN14604949	JABDZY000000000	4.91	19	4505	4570	53.41	Toombs Co.	+	Onion
PNA_07_1	SAMN14604950	JABDZX000000000	4.92	33	4492	4557	53.47	Tattnall Co.	+	Onion
PNA_06_4	SAMN14604951	JABDZW000000000	4.96	49	4557	4620	53.6	Wayne Co.	+	Onion
PNA_05_1	SAMN14604952	JABDZV000000000	5.07	41	4655	4726	53.35	Vidalia Region	+	Onion
PNA_03_2	SAMN14604953	JABDZU000000000	5.06	108	4626	4698	53.51	Tift Co.	−	Onion
PNA_03_1	SAMN14604954	JABDZT000000000	4.96	27	4588	4659	53.55	Tift Co.	+	Onion
PNA_02_12	SAMN14604955	JABDZS000000000	4.73	34	4300	4373	53.58	Tift Co.	+	Onion
PANS_99_9	SAMN14604956	JABDZR000000000	5.1	61	4684	4759	53.53	Tift Co.	−	Prairie verbena
PANS_99_5	SAMN14604957	JABDZQ000000000	4.99	46	4612	4683	53.51	Tift Co.	−	Prairie verbena
PANS_99_4	SAMN14604958	JABDZP000000000	5.12	39	4654	4719	53.29	Tift Co.	+	Florida pusley
PANS_99_36	SAMN14604959	JABDZO000000000	4.87	41	4458	4524	53.5	Terrell Co.	−	Florida pusley
PANS_99_33	SAMN14604960	JABDZN000000000	4.98	37	4553	4621	53.28	Coffee Co.	+	Florida pusley
PANS_99_32	SAMN14604961	JABDZM000000000	4.99	28	4576	4653	53.53	Vidalia Region	−	Florida pusley
PANS_99_31	SAMN14604962	JABDZL000000000	5.67	938	5105	5234	52.63	Tattnal Co.	+	Texas millet
PANS_99_29	SAMN14604963	JABDZK000000000	4.86	37	4447	4516	53.46	Tift Co.	+	Crab grass
PANS_99_27	SAMN14604964	JABDZJ000000000	5.35	621	4720	4797	53.54	Vidalia Region	+	Florida beggarweed
PANS_99_26	SAMN14604965	JABDZI000000000	4.95	33	4481	4559	53.17	Vidalia Region	−	Hyssop spurge
PANS_99_25	SAMN14604966	JABDZH000000000	4.83	24	4418	4487	53.54	Vidalia Region	+	Bristly starbur
PANS_99_23	SAMN14604967	JABDZG000000000	4.94	32	4527	4597	53.3	Vidalia Region	−	Yellow nut sedge
PANS_99_22	SAMN14604968	JABDZF000000000	4.96	27	4570	4638	53.55	Tift Co.	−	Crab grass
PANS_99_11	SAMN14604969	JABDZE000000000	5.08	46	4698	4767	53.22	Tift Co.	+	Crab grass
PANS_4_2	SAMN14604970	JABDZD000000000	4.91	30	4498	4564	53.52	Tift Co.	−	Adult tobacco thrip from peanut
PANS_200_2	SAMN14604971	JABDZC000000000	4.9	113	4418	4489	53.39	Reidsville	+	Pink purslane
PANS_200_1	SAMN14604972	JABDZB000000000	4.83	20	4429	4498	53.22	Reidsville	−	Slender amaranth
PANS_2_8	SAMN14604973	JABDZA000000000	4.84	28	4462	4531	53.41	Tift Co.	+	Thrips infected peanut leaf
PANS_2_7	SAMN14604974	JABDYZ000000000	4.84	28	4466	4534	53.42	Tift Co.	+	Thrips from peanut blossoms
PANS_2_6	SAMN14604975	JABDYY000000000	5.1	31	4682	4747	53.44	Tift Co.	+	Thrips from peanut blossoms
PANS_2_5	SAMN14604976	JABDYX000000000	4.86	39	4468	4534	53.41	Tift Co.	+	Thrips from peanut blossoms
PANS_2_1	SAMN14604977	JABDYW000000000	5.01	38	4610	4679	53.55	Tift Co.	−	Adult tobacco thrip from peanut
PANS_1_9	SAMN14604978	JABDYV000000000	4.96	22	4576	4647	53.51	Tift Co.	−	Thrip feces from peanut leaf
PANS_1_8	SAMN14604979	JABDYU000000000	5	31	4596	4664	53.45	Tift Co.	−	Adult tobacco thrip
PANS_1_6	SAMN14604980	JABDYT000000000	4.81	21	4395	4466	53.45	Tift Co.	+	Adult tobacco thrip
PANS_1_5	SAMN14604981	JABDYS000000000	4.81	23	4398	4476	53.45	Tift Co.	+	Adult tobacco thrip
PANS_1_2	SAMN14604982	JABDYR000000000	4.92	32	4520	4590	53.48	Tift Co.	+	Thrips from onion leaf
PANS_1_10	SAMN14604983	JABDYQ000000000	4.96	26	4578	4651	53.51	Tift Co.	−	Thrip feces from peanut leaf

*‘+’ indicates red-onion scale necrosis causing strain ‘-’ indicates non-red-onion-scale necrosis causing strain.

Inoculum was prepared by transferring single colonies of each bacterial strain from 48-h-old cultures on nutrient agar (NA) medium to nutrient broth (NB). The broth was shaken overnight on a rotary shaker (Thermo Fisher Scientific, Gainesville, FL, United States) at 180 rpm. After 12 h of incubation, 3 ml of each bacterial suspension were centrifuged at 6,000 × *g* (Eppendorf, Westbury, NY, United States) for 2 mins. The supernatant was discarded and the pellet was re-suspended in sterile water. Inoculum concentration was adjusted using a spectrophotometer (Eppendorf, Westbury, NY, United States) to an optical density of 0.3 at 600 nm [≈1 × 10^8^ colony forming unit (CFU)/ml]. The bacterial suspension was diluted serially in sterile distilled water to obtain the desired concentration.

### *Pantoea ananatis* Phenotyping: Red Onion Scale Necrosis Assay as a Measure of Onion-Pathogenicity

The red onion scale necrosis assay was conducted as described by Stice et al. (2018). Briefly, red onion bulbs (cv. Red Creole) were each surface-disinfested by removing the outer, dry scales and then spraying the outer exposed fleshy scales with a 3% sodium hypochlorite solution using a spray bottle. This was followed by wiping the outer scales dry with a sterile paper towel. Later, the bulbs were sprayed with sterile distilled water and wiped again with a sterile paper towel. Surface-disinfested onion scales were each sliced into approximately 6 cm × 3 cm (length × width) segments using a surface-disinfested knife. Each onion scale piece was pierced on the inner surface with a pipette tip, and 10 μl of the relevant bacterial suspension (1 × 10^6^ CFU/ml) was deposited on the wounded tissue. Scales were maintained in petri plates containing autoclaved paper towels moistened with sterile water. Petri plates were kept in an aluminum tray covered with a plastic lid. The inoculated onion scales were incubated at room temperature for 96 h in the dark, after which the size of the necrotic, pigment-clearing zone was recorded as a measure of pathogenicity. Strains that cleared the red anthocyanin pigment and caused necrosis were classified as pathogenic, and those that did not were classified as non-pathogenic to onion (Supplementary Figure 1). Three replicates of onion-scale pieces were used to test each strain, and the experiment was repeated twice (a total three experiments). Onion scales inoculated with sterile water and a known pathogenic strain of *P. ananatis* (PNA 97-1) served as negative and positive control treatments, respectively.

To confirm that symptoms on the onion scale were caused by *P. ananatis*, bacteria from symptomatic scale tissue (*n* = 3) were isolated from the margins of the necrotic area and healthy tissue, and streaked onto Tryptic soy broth agar (TSBA) and incubated for 48 h at 28°C. Yellow-pigmented colonies were selected to test for genus and species identity using physiological tests and a species-specific TaqMan-based polymerase-chain reaction (PCR) assay (Dutta et al., 2014) for *P*. *ananatis*. Briefly, presumptive *P. ananatis* colonies were picked using a sterile inoculation loop and suspended separately in 2 ml micro-centrifuge tubes, each containing 25 μl of sterile deionized water. The bacterial suspension was heated (Modular Dry Block Heaters, Cole Parmer, IL, United States) for 3 min at 95°C. A suspension (5 μl) was amplified in 20 μl of PCR master-mix containing 10 mMmTris-HCl (pH 9.0), 50 mM KCl, 0.1% Triton X-100, 1.5 mM MgCl_2_, and 0.2 mM of each nucleotide (dATP, dCTP, dGTP, and dTTP), 25 μM each of primer PanITS1 (5′-GTCTGATAGAAAGATAAAGAC-3′) and EC5 (5′-CGGTGGATGCCCTGGCA-3′) and 10 μM of TaqMan probe 6-FAM TAGCGGTTAGGACTCCGCCCTTTCA-BHQ. The PCR reaction was conducted in a Cepheid Smart Cycler (Sunnyvale, CA, United States) using the following thermal profile: denaturation at 95°C for 180 s, 35 cycles each of denaturation at 95°C for 15 s, and annealing at 60°C for 40 s. Samples with cycle threshold (Ct) values <35 were considered positive for *P. ananatis*.

### DNA Isolation and Library Preparation for Whole Genome Sequencing of *P. ananatis*

*Pantoea ananatis* strains (*n* = 81) were revived from the 15% aqueous glycerol solution at −80°C by streaking individually onto NA and incubated for 48-h at 28°C. After incubation, a single colony of each strain was picked and placed into 3 ml of Luria Bertani broth. The resulting broth was shaken overnight on a rotatory shaker (Thermo Fisher Scientific, Gainesville, FL, United States) at 180 rpm at 30°C. After incubation, broth (1.5 ml) cultures were each centrifuged in a microcentrifuge tube (2 ml) at 6,000 × *g*. The supernatant was discarded and the bacterial pellet suspended in 1 ml of sterile water, from which DNA was isolated. DNA isolation was done using a DNeasy ultra clean microbial DNA extraction kit (Qiagen, Germany) using the manufacturer’s instructions. DNA samples were quantified (ng/μl) using a Nanodrop (Thermo Fisher Scientific, Gainesville, FL, United States).

For Illumina Nextera library preparation, a total of 100 ng DNA from each bacterial strain was used according to KAPA Hyper Prep kit (KAPA Biosystems, MA, United States) at the Georgia Genomics and Bioinformatics Core (GGBC), University of Georgia, Athens, GA, United States. Briefly, the genomic DNA from each bacterial strain was fragmented followed by end repairing and A-tailing, which produced end-repaired 5′-phosphorylated, 3′-dA-tailed DNA fragments. Adapters were ligated resulting in a 3′-dTMP overhang ligated to 3′-dA-tailed molecules. Post-ligation cleanup was performed to remove un-ligated adapter and/or adapter-dimer molecules from the library before library amplification. Library amplification was done employing a high-fidelity, low-bias PCR assay to amplify library fragments with appropriate adapters on the ends. Dual indexing was done during the library preparation and PCR amplification. Dual indexing (by introducing indexes into both library adapters) was done to overcome the occurrence of mixed clusters on the flow as this is a predominant source of error while multiplex sequencing. Libraries for the 81 bacterial strains were pooled and sequenced on the Illumina Nextseq500 using a high output run. All samples were sequenced to produce 150 bp paired end reads.

### Read Data Filtering

FastQC was run to assess the raw fastq files. Overall, data quality was good with typical drop off in quality at the ends of the reads. The number of raw reads ranged from 8.4 million to 18.2 million PE reads. The read data were filtered to remove low quality reads/bases and trimmed for reads containing primer/adaptor sequences using Trimmomatic (v 0.36) in paired end mode (Bolger et al., 2014). Further, all 5′ and 3′ stretches of ambiguous ‘N’ nucleotides were clipped to ensure high quality reads for downstream analysis. Trimmed data were re-assessed using FastQC. These data were used for genome assembly followed by pan-genome analyses. A total of 675.6 million raw reads were generated and, after stringent quality filtering, 657.6 million high-quality reads were obtained including a lowest of 3.55 million and a highest of 16.4 million reads (Supplementary Table 1).

### Genome Assembly and Pan-Genome Analyses

Trimmed reads were assembled using the SPAdes (v 3.11.1) assembler (Bankevich et al., 2012). Both the paired and unpaired data were used in assembly at default settings. Assembly files were submitted to NCBI under the bio-project identity PRJNA624643. The assembled contigs were also submitted to LINbase to obtain life identification numbers (LIN) (Supplementary Table 18). The scaffolds of the respective 81 *P. ananatis* strains were annotated using prokka (v 1.13) (Seemann, 2014) to produce gff3 (general feature format) and gbk (gene bank format) files. The gbk files were used for pan-genome analyses by using get_homologues (Contreras-Moreira and Vinuesa, 2013). These gbk files were used to get the syntenic sequence clusters by get_homologues.pl using OrthoMCL (OMCL) algorithm. In order to check the openness/closeness of the pan-genome, the theoretical estimation of pan-genome size was carried out using an exponential model of Tettelin et al. (2005), which was fitted to the OMCL accessory gene clusters. The syntenic clusters generated were used to a develop a pan-genome matrix showing presence and absence variants (PAVs) using compare_clusters.pl, and the pan-genome matrix was used to classify the genes into core, soft-core, shell and cloud genes using parse_pangenome_matrix.pl (auxiliary script of get_homologues.pl). Core genes (part of soft-core) were defined as those present in all 81 genomes and accessory genes were present in a subset of the 81 strains. The accessory gene cluster was further divided into shell and cloud gene clusters. Soft-core genes occurred in 95% of the genomes. Cloud genes were present in ≤2 genomes and shell genes comprised of remaining genes (Contreras-Moreira and Vinuesa, 2013). Distribution of cluster sizes as a function of the number of genomes these clusters contained was displayed using R with parse_pangenome_matrix.pl. Gower’s distance matrix was generated using the tab delimited pan-genome PAV file as input by executing the shell script hcluster_pangenome_matrix.sh (auxiliary script of get_homologues) when used to call R function hclust. The presence and absence of 14 HiVir/PASVIL and 11 *alt* cluster genes were determined by looking at the cluster of genes using compare_clusters.pl program and blastn search, respectively. HiVir/PASVIL genes that were clustered from different strains were considered present in those strains. Each of the *alt* cluster sequence was subjected to blastn against each of the 81 genome assemblies. If a blast hit was found against a genome, that gene was considered present. The *alt* cluster was considered conserved in a given strain if all 11 genes were present, and was considered absent if anywhere from 9 to 11 genes were absent. Similarly, if all 14 genes of the HiVir/PASVIL cluster were present in a strain, then it was considered conserved, otherwise it was rated as absent if all some or all of the genes were absent in a particular strain. The presence and absence of these genes was plotted as heat maps.

### Horizontal Gene Transfer (HGT) and Prediction of Genomic Islands, and Pathogenicity and Symbiotic Factors in *P. ananatis*

The horizontal gene transfer (HGT) study was conducted in two steps. Broadly, the first step involved phylogenetic classification of genomes based on conserved genes to identify the closely clustered genomes. The second step used the genomes that clustered together in groups (A-E) to find the HGT events among these groups. In the first step, all *P. ananatis* draft genomes were scanned for the presence of 120 conserved bacterial marker genes or single copy genes (SCG) using GTDB toolkit (Chaumeil et al., 2020). Genomes were assigned to the domain with the highest proportion of identified marker genes. The selected domain-specific markers were aligned with HMMER, concatenated into a single multiple sequence alignment (MSA). Based on MSA, strains (81 genomes) were classified into a reference phylogenetic tree. The tree file was visualized in iTOL^1^. The resulted tree was resolved into five groups based on phylogenetically closely related genomes that grouped in the same cluster. In the second step, these grouped genomes were used as input for MetaCHIP (Song et al., 2019) algorithm to identify donor and recipient genes among the customized user defined phylogenetic groups (Figures 3A,B). MetaCHIP identified putative donor and recipient transfer events within the 81 *P. ananatis* strains based on combined similarity and phylogenetic incongruency (Douglas and Langille, 2019).

We used *P. ananatis* strains (PNA_99_3 and PNA_99_14) to identify the genomic islands, and pathogenicity and symbiotic factors in respective genomes as they shared maximum number of HGT events (*n* = 67). SIGI-HMM and IslandPath-DIMOB prediction methods in IslandViewer 4 was used to predict the genomic islands (Bertelli et al., 2017). GIPSy was used to predict the pathogenicity and symbiotic factors (Soares et al., 2016).

### Presence and Absence Variations, Core Genome SNPs, and Whole Genome Multi Locus Sequence Typing (wgMLST) Based Phylogeny

To carry out the phylogenetic analysis of *P. ananatis* strains (*n* = 81), PAVs and core SNPs were identified using Panseq pipeline (Laing et al., 2010). Core genome threshold of 81 and sequence identity of 85% was used. SNPs identified within the shared core genome regions were obtained in Phylip multiple sequence alignment format. PAVs were identified in a binary Phylip format. Phylogenetic trees using PAVs and SNPs were constructed using RAxML (Stamatakis, 2014). RAxML with rapid bootstrapping returned the best ML tree with support values after 1000 bootstrap searches. PAVs- and SNPs-based phylogenetic analysis involved 81 sequences with 16,190 and 2112 positions, respectively in the final dataset in each sequence. PAVs along with SNPs were identified using pan-seq to conduct comparative phylogenetic analysis. Further, whole genome multi locus sequence typing (wgMLST) tree was constructed (Liu et al., 2016). To carry out this analysis the assembled contigs file of each of the 81 *P. ananatis* strains were uploaded to PGAdb-builder and a pan-genome allele database was constructed. This database was used to build wgMLSTtree using PGAdb-builder as described earlier (Liu et al., 2016).

### Average Nucleotide Identity (ANI)/Average Amino Acid Identity (AAI), Pan-Genome Wide Association and Annotation

Get_homologues perl program was used to estimate the average nucleotide (ANI) and amino acid (AAI) identities of CDSs and the proteins coded by the CDSs among all individual strains of the pan-genome. The resulting ANI (-a ‘CDS’) and AAI pan-genome matrices obtained using get_homologues.pl were used to plot the heat maps with the shell script plot_matrix_heatmap.sh. For AAI, BLASTP scores were calculated among protein sequences. For association analysis, the pan-genome matrix was used with the phenotyping data using Scoary, a python program (Brynildsrud et al., 2016). Scoary was used to calculate associations among genes in the pan-genome and the red-scale necrosis assay (a qualitative; pathogenic- vs. non-pathogenic association). The output of this program comprised of a list of genes sorted by strength of association with these traits. Genes with a naïve *p*-Value < 0.05, a Bonferroni *p*-Value < 0.05 and corrected *p*-Value (Benjamini-Hochberg) of association < 0.05 were considered significant. The predicted core, soft-core, shell and cloud gene sequences were annotated by conducting a blastx search (*E*-value ≤ 1e-5) against the NR database. The blast output was generated in an XML format that was used as input for Blast2GO to assign gene ontology (GO) terms and assignment of KEGG pathways (Conesa and Götz, 2008). Based on the assigned GO categories each component of pan-genome was categorized into biological process (BP), molecular function (MF) and cellular component (CC).

## Results

### The *P. ananatis* Genome and Pan-Genome Architecture

Overall, more than 97% of the read data were retained after quality filtering, amounting to 98.6 Gb (Supplementary Figure 2). These quality-filtered reads were used to construct draft assemblies. We conducted pan-genome analyses on the draft genomes of the 81 selected *P. ananatis* strains collected from onions, weeds and thrips from different regions of Georgia, United States (Table 1). The size of the draft assemblies ranged from 4.7 to 5.8 Mb. The strains PNA 02-12 and PNA 98-11 had minimum and maximum assembly sizes of 4.7 and 5.8 Mb, respectively (Table 1). In all the assemblies, the total number of protein-coding sequences ranged from 4,300 to 5,110, and the number of mRNAs ranged from 4,373 to 5,234 (Table 1).

The full spectrum of the pan-genome contained 14,452 protein coding genes. Among these, 3,799 genes including 3,153 genes present in all 81 strains (core) and 646 genes present in ≥76 genomes <81 genomes defined the soft-core. The largest group of 6,808 genes (≤2 genomes) were cloud and the remaining 3,845 genes belonged to shell (≤3 and =75 genomes) gene cluster (Supplementary Figures 3A,B). Cloud genes are considered to be unique genes contributed by each strain of *P. ananatis*. In this study, we found 6,808 genes that are unique to two or less genomes. Details of the number of core and accessory genes contributed by each strain are listed in Supplementary Table 2.

The ANI and AAI are widely practiced genome-based characteristics based on pairwise genome comparisons and averaging the sequence identities of shared orthologus genes (amino acids for AAI and nucleotide for ANI) and are determinant for the circumscription of prokaryotic species (Rosselló-Mora, 2005). We, therefore, estimated ANI- and AAI-based all vs. all matrices and constructed clustered tree-based heat-maps. Overall, the ANI and AAI among 81 strains varied from 99.0 to 99.9% and 96.0 to 99.8%, respectively, which confirmed that all strains used in this study belonged to the species *P. ananatis* (Supplementary Figure 4).

### Phenotyping

All the strains used were identified as *P. ananatis* based on PCR assays, prior to phenotyping. *Pantoea ananatis* strains were classified as pathogenic and non-pathogenic based on the ability to necrotize red onion-scales and clear the anthocyanin pigment. Strains that caused red onion scale necrosis and cleared the anthocyanin pigment were classified as pathogenic (+) and those that did not were identified as non-pathogenic (−) (Table 1). Based on the red-scale necrosis assay, 62.9% (*n* = 51) and 37.1% (*n* = 30) of the strains were identified as pathogenic and non-pathogenic, respectively. Among the strains that were isolated only from onions (regarded as PNA), 68% (36 of 53 onion strains) were able to cause red-onion scale necrosis (Table 1). In contrast, among the strains (*n* = 28) that were isolated from weeds and thrips (regarded as PANS), 28.5% (8 of 28) and 25% (7 of 28) were able to cause necrosis on the red-onion scale, respectively. A majority of strains (13 of 28) from non-onion sources [weeds: 28.5% (8 of 28) and thrips: 17.8% (5 of 28)] were classified as non-pathogenic as they were not able to cause red-onion scale necrosis (Table 1; Supplementary Figure 1).

### *Pantoea ananatis* Has an Open Pan-Genome

**FIGURE 1:**
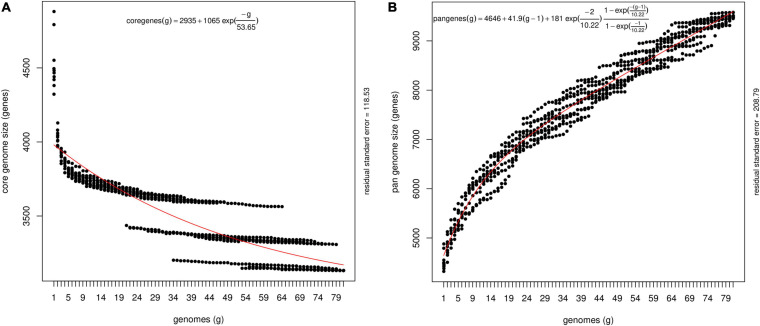
Theoretical estimation of the core and pan-genome sizes based on the exponential decay model. **(A)** Estimation of core genome size based on Willenbrock model fit to OMCL clusters. The equation for the core gene estimation include theoretical estimate of the number of core genes (2395), free parameters for amplitude of exponential decay (1065) and decay constant (53.65). Decay constant measures the speed at which the core genes converges to its asymptotic value. Size of the core genome is shown here as ‘2395’ for the number of genomes →∞, where number of genomes display continuous extrapolation of the number of strains considered. The core genome, that is, the number of conserved genes present in all genomes, was estimated by fitting an exponential decay function by non-linear least squares. **(B)** Estimation of pan-genome size based on Tettelin model fit to OMCL clusters. The equation for the theoretical estimation of pan-genome include average number of genes per sequenced genome (4646). The other components in the equation depict a linear term [41.9 (g-1)] that represents extrapolated angle of growth of the size of pan-genome size without introducing new free parameters, amplitude of exponential decay (181), and decay constant (10.88).

The pan-genome architecture of the 81 *P. ananatis* genomes analyzed is characterized in Figure 1. We used the exponential decay model of Willenbrock et al. (2007) that fitted the core gene clusters generated using the OMCL algorithm, which predicted a theoretical core genome of 2,935 genes. In addition, the core genome was not continuous because of the draft assemblies (not the finished sequence assembly) used in this study (Figure 1A). In order to check the openness/closeness of the pan-genome, the theoretical estimation of pan-genome size was carried out using an exponential model of Tettelin et al. (2005), which was fitted to the OMCL accessory gene clusters. The pan-genome samples appeared to converge to linear growth with >10,000 genes, with ∼50 new genes being added on average to the pan-genome with each new *P. ananatis* genome sequenced (Figure 1B). Our results indicate that the pan-genome of *P. ananatis* is open.

### Analysis of *P. ananatis* Strains Revealed Pathogenic and Non-pathogenic Differentiation

**FIGURE 2:**
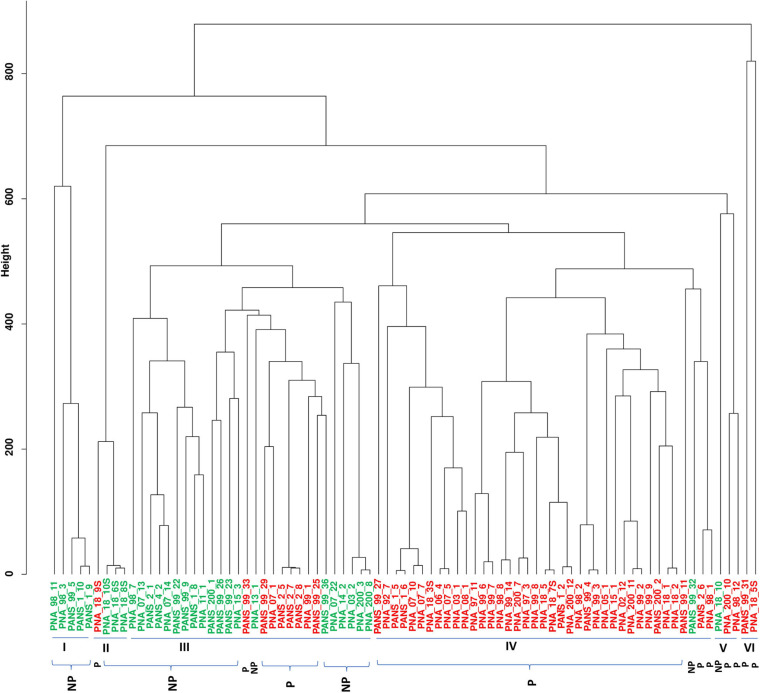
Dendrogram of 81 pathogenic and non-pathogenic strains of *Pantoea ananatis* based on the soft-core genes. Dendrogram to detect outliers with cut-off height is shown in the y-axis. The height scale is the distance metrics between the clusters, and strains displaying low merging height are highly related. Strains highlighted in green are non-pathogenic (NP) and ones in red are pathogenic (P). Bacterial strains of onion and non-onion origin are designated with a prefix as “PNA” and “PANS”, respectively.

We used 51 onion pathogenic and 30 non-pathogenic strains as identified using the red-scale necrosis assay. The pathogenic strains displayed typical scale-clearing phenotype as opposed to no symptoms observed in case of non-pathogenic strains (Supplementary Figure 1). Based on pan-genome enabled phylogenetic-tree, strains were distributed in six clusters. Further, we also constructed dendrograms using only shell and cloud PAVs and compared the clustering of pathogenic- and non-pathogenic strains. A soft-core genome based (including 3153 conserved core genes and 646 genes present in ≥76 genomes) dendrogram was constructed to understand the relationship among the *P. ananatis* strains (Figure 2). Soft-core genes resulted in six clusters with cluster I, II and III mainly consisting of non-pathogenic (colored green in figure) strains. Cluster III contained eight pathogenic (colored red in figure) out of total 24 strains. Cluster IV comprised of all pathogenic strains except for the presence of one non-pathogenic strain. Cluster V and VI comprised of mainly pathogenic strains with one exception (one non-pathogenic strain was present in cluster V) (Figure 2).

Clustering using shell PAVs resulted in five broad clusters; cluster I with all 7 pathogenic strains, cluster II- with five non-pathogenic (green) strains, cluster III with all 23 pathogenic (red), cluster IV with 17 pathogenic (red) out of total 25 strains and cluster V with four pathogenic (red) out of the total 21 strains (Supplementary Figure 5). Cloud PAVs showed a mixed pattern of clustering of pathogenic- and non-pathogenic strains (Supplementary Figure 6).

### Identification of Horizontal Gene Transfer (HGT), Genomic Islands, and Pathogenicity and Symbiotic Factors in *P. ananatis*

**FIGURE 3:**
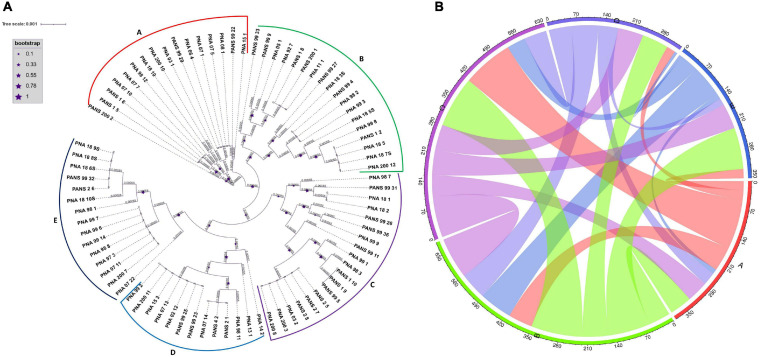
Phylogeny based horizontal gene transfer among 81 *Pantoea ananatis* strains. **(A)** Phylogenetic tree of 81 *P. ananatis* strains based on multiple sequence alignment of 120 single copy genes. Phylogenetic tree resulted in five clusters **(A–E)**. Size of stars represents the bootstrap values in that order. **(B)** Predicted gene flow within the five phylogenetic clusters of *P. ananatis*. Bands connect donors and recipients, with the width of the band correlating to the number of HGTs and the color corresponding to the donors. Numbers on the circumference of circos plot represent the number of genes that undergo horizontal gene transfers. The alphabets on five arcs **(A–E)** of circos represent the five phylogenetic clusters of *P. ananatis* strains.

Phylogenetic analyses based on multiple sequence alignment resulted in five predominant clusters (cluster A-E) (Figure 3). Groupings were assigned to each genome based on the phylogenetic classification. Further, based on the assigned groupings, HGT study was conducted to assess the transfer of genes from putative donor strains to putative recipient strains (Figure 3 and Supplementary Table 3) to the putative recipient strains. The HGT events in *P. ananatis* were found to be extensive. We found 1,182 HGT events among the 77 strains with 68 putative donor and 70 recipient strains (Supplementary Table 3). A maximum of 114 putative gene transfers occurred from PNA_98_12 (cluster A) followed by 106 from PNA_99_3 (cluster B), 61 from PANS_99_9 (cluster B), and 60 from PNA_07_22 (cluster E) (Figure 3 and Supplementary Table 3). Rest of the HGTs between genomes ranged from 1 gene to 30 genes (Supplementary Table 3). Three strains that received the greatest number of gene transfers were: PNA_98_3 (cluster C) that received 125 gene transfers, followed by 84 genes received by PNA_99_14 (cluster E) and 64 genes received by PANS_99_4 (cluster B) (Figure 3 and Supplementary Table 3). Highest number of 67 HGTs occurred from PNA_99_3 to PNA_99_14 followed by 32 HGTs from PNA_98_12 to PNA_98_3 and 31 HGTs from PNA_99_3 to PNA_98_3. Notably, the maximum number of HGT events occurred between the donor and the recipient strains that were identified in the years 1997, 1998 and 1999. Out of the total 114 genes transferred by PNA 98_12, a maximum of 32 were received by PNA 98_3 followed by PNA_97_11 (21 genes). Similarly, for the second highest donor PNA_99_3, out of 106 HGT events, 67 were transferred to PNA_99_14 and 31 to PNA_98_3. However, PNA_07_22 with third most number (60) of donor genes had PANS_99_4 as the recipient with most number (31) of HGTs.

Four genes of the HiVir/PASVIL cluster were found to be horizontally transferred between the strains. These genes were flavin-dependent oxidoreductase (*pavC*), SAM-dependent methyltransferase (*pavG*), GNAT family N-acetyltransferase (*pavH*) and MFS transporter (*pavJ*). Flavin-dependent oxidoreductase (*pavC*) was transferred from PNA_200_3 (00224) and PNA_200_8 (00224) to PNA_98_11 (01052), *pavG* was transferred from PNA_99_1 (01588) and PNA_99_8 (02166) to PNA_05_1 (01633) and PNA_99_14 (02553) respectively. In case of *pavH*, 25 HGTs were identified and for *pavJ*, a total of 16 HGTs were identified. Another homologous HiVir/PASVIL gene involved in analogous tricarboxylic acid cycle reaction was homocitrate synthase that was involved in HGT between PNA_99_3 (01851) and PNA_99_12 (02485) (Supplementary Table 4).

We further investigated the *alt* cluster genes to be involved in HGT. Seven *alt* genes namely, *altA* (alkene reductase), *altB* (SDR family oxidoreductase), *altC* (DsbA family oxidoreductase), *altE* (carboxymuconolactone decarboxylase family protein), *altH* (DNMT family transporter), *altI* (aminotransferase) and *altR* (TetR/AcrR family transcriptional regulator) were horizontally transferred between strains. Six HGTs were identified for *altH*, five for *altB*, two each for *altE* and *altI* and one each *altA*, *altE* and *altR* (Supplementary Table 4).

We used *P. ananatis* strains, PNA_99_3 and PNA_99_14 to identify the genomic islands, and pathogenicity and symbiotic factors in respective genomes. These two strains were selected as they shared maximum number of HGT events (*n* = 67). In PNA_99_3, SGI-HMM and IslandPath-DIMOB predicted a total of 25 (length = 4.3 kb) and 14 (length = 197.2 kb) genomic islands, respectively (Supplementary Tables 5-8). In case of PNA_99_14, SGI-HMM and DIMOB predicted 21 (length = 4.06 kb) and 13 (length = 210.6 kb) genomic islands, respectively (Supplementary Tables 9-12). A total of 380 pathogenicity and 150 symbiotic factors were identified in PNA_99_3 (Supplementary Tables 5-8) and 410 pathogenicity and 169 symbiotic factors were identified in PNA_99_14 (Supplementary Tables 9-12). Among several genes present in the genomic islands and, pathogenicity and symbiotic factors, HiVir and *alt* cluster genes were identified. HiVir cluster genes that were identified include 3-isopropylmalate dehydratase large sub-unit (*pavE*), 3-isopropylmalate dehydratase small sub-unit (*pavF*), nitrilotriacetate monooxygenase (flavin monooxygenase) (*pavC*), SAM dependent methyltransferase (*pavG*), MFS transporter (*pavJ*) phosphoenolpyruvate mutase (*pepM*), as a part of genomic islands in both PNA_99_3 and PNA_99_14 (Supplementary Tables 5, 6, 9, 10). Similarly, two *alt* cluster genes were identified in the predicted pathogenicity factors. The *alt* genes identified were tetR family transcriptional regulator (*altR*) in PNA_99_14 (Supplementary Table 11) and thioredoxin reductase (*altD*) in PNA_99_3 and PNA_99_14 (Supplementary Tables 7,11).

### Presence and Absence Variations, Core Genome SNPs, and Whole Genome Multi Locus Sequence Typing (wgMLST) Based Phylogeny

**FIGURE 4:**
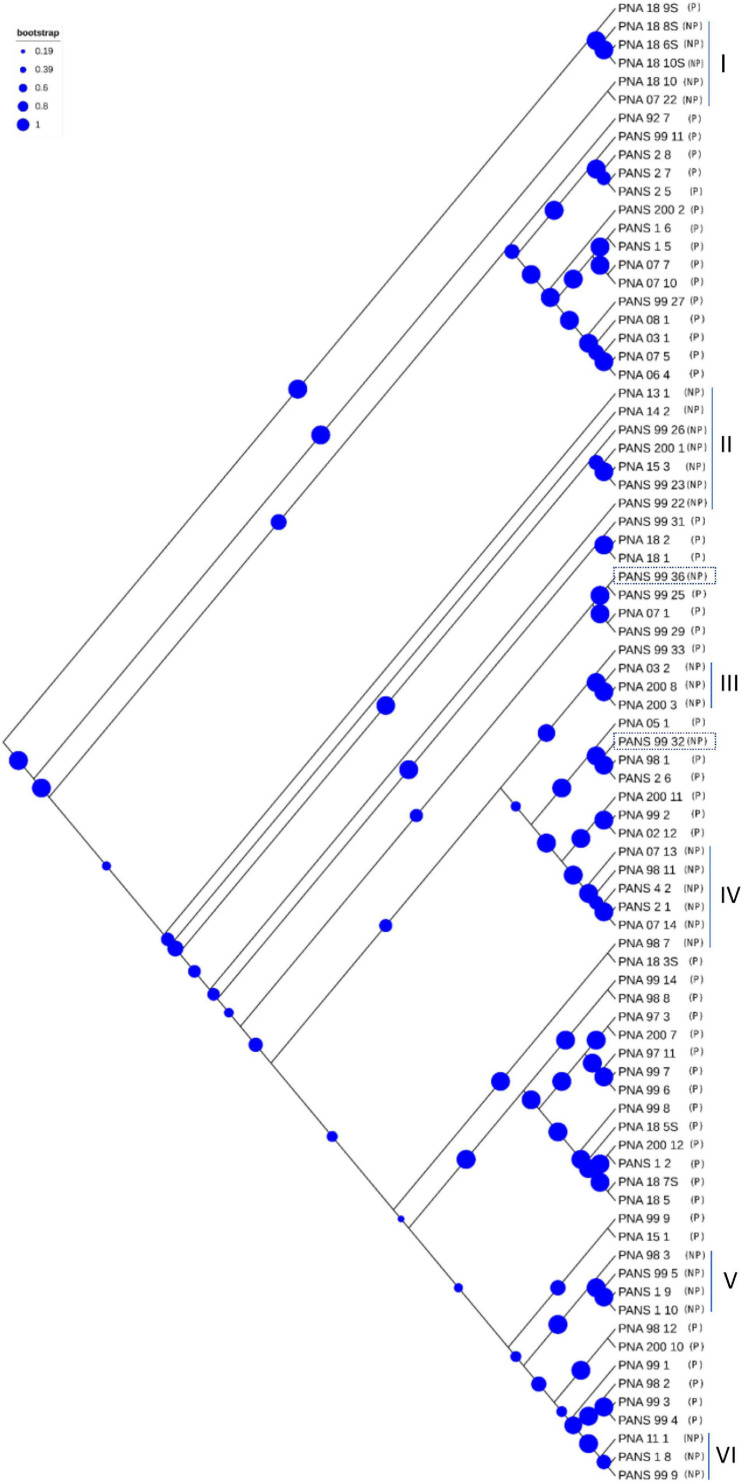
Phylogenetic tree based on whole genome multi-locus sequence typing (WgMLST). Dendrogram for 81 *Pantoea ananatis* strains was constructed using assembled genome contigs. The letter ‘P’ represents pathogenic and ‘NP’ represents non-pathogenic strains. Strains labeled as ‘PNA’ represent onion as a source of isolation whereas strains labeled as ‘PANS’ represent source of isolation other than onion. Size of blue circles represent the bootstrap values. Six ‘NP’ clusters that grouped separately from the ‘P’ strains are numbered as I to VI and the two NP strains that grouped with ‘P’ strains are highlighted in rectangular blocks.

The PAVs were identified using Pan-seq pipeline along with SNPs to carry out a comparative phylogenetic analysis based on PAVs and SNPs. PAVs-based phylogeny as compared to core-SNPs-based phylogeny distinguished the pathogenic strains (P) from the non-pathogenic (NP) strains (Supplementary Figure 7). Considering the strictest constrain of 81 genomes, SNPs were identified that were present in all genomes used in this study. The 51 pathogenic and 30 non-pathogenic strains were distributed in several groups with both pathogenic and non-pathogenic strains grouped together (Supplementary Figure 7A). However, PAVs-based phylogenetic tree showed that the 30 non-pathogenic strains clustered separately from the pathogenic strains in three groups of 16, five and nine strains. Largely, the strains were separated out based on their pathogenicity in PAVs-based phylogeny. SNPs- and PAVs-based phylogenetic analysis identified PNA_98_11 (NP), PNA_98_12 (P), PANS_99_27 (P), and PNA_92_7 (P) as the most diverse strain based on its branch length. PAVs-based phylogeny identified PNA_98_1 (P) and PNA_18_10 (NP) as other diverse strains that were not as diverse with SNPs-based phylogenetic analysis (Supplementary Figures 7A,B). We also used wgMLST based approach to construct a phylogenomic tree using the assembled scaffolds of 81 *P. ananatis* strains. In this approach first, a pan-genome allele database for *P. ananatis* strains were established using PGAdb-builder. The database consisted of 3,370 alleles in 81 *P. ananatis* strains. Using this allelic distribution, a genetic relatedness tree was constructed (Figure 4). There were six different clusters of non-pathogenic (NP) strains that were grouped separately from the pathogenic (P) strains. However, two non-pathogenic strains (PANS_99_36 and PANS_99_32) grouped arbitrarily with pathogenic strains (Figure 4).

### Pan-Genome-Wide Association Study

Presence and absence of each candidate genes in the accessory genomes was screened and scored. Further, Scoary was used to identify genes that were significantly associated with red-onion scale necrosis (indicative of pathogenicity to onion) using the 81 pathogenic- and non-pathogenic *P. ananatis* strains. Scoary predicted 42 genes, including the 14 HiVir/PASVIL cluster genes (*hvaA*, *pepM, pavC-pavN*) that are shown to be associated strongly with red-onion scale necrosis or pathogenicity to onion based on stringent *p*-Values (Supplementary Table 13). Earlier the same cluster of HiVir genes was predicted to be responsible for onion pathogenicity (Asselin et al., 2018; Takikawa and Kubota, 2018). A total of 28 genes were identified outside the HiVir/PASVIL cluster. Eight of the 28 genes are annotated and the remaining 20 are hypothetical. Annotated functions of eight genes include: site-specific tyrosine recombinase (*xerD6*), pyridoxal-4-dehydrogenase (*pld_1*), murein tetrapeptide carboxypeptidase, sporulation initiation inhibitor protein (*soj3*), N-acetylmuramoyl-L-alanine amidase (*amiD_4*), conjugal transfer protein (*traR_3*), adhesin/invasin TibA autotransporter (*tibA*), and helix turn helix-type transcriptional regulator (*dmlR_11*) (Supplementary Table 11). Out of the 20 hypothetical genes, 13 genes (four, five and four genes) could possibly be constituting separate operons in *P. ananatis* because of their contiguity (Supplementary Table 13 highlighted in colors blue, green and red).

Out of 14 HiVir/PASVIL cluster genes associated with pathogenicity to onion, two are annotated as hypothetical (*hvaA* and *pavK*) and the remaining 12 are annotated with functions in metabolite production (Table 2). These 12 genes include *pepM*, coding for phosphonopyruvate mutase; *pavC*, that encodes nitrilotriacetate monooxygenase component A (catalyzes plant-derived aromatic compounds); *pavD* for homocitrate synthase; two genes associated with leucine biosynthesis associated metabolites, including *pavE* for 3-isopropylmalate dehydratase large subunit and *pavF* for 3-isopropylmalate dehydratase small subunit; *pavG*, which codes for SAM dependent methyltransferase; *pavH* for N-acetyltransferase; *pavI* for the ATP-grasp domain containing protein; *pavJ* for MFS transporter; *pavL* which encodes flavin reductase; *pavM* for carboxylate-amine ligase; and *pavN* as transposase.

**TABLE 2:** Highest-ranking genes associated with red-onion scale necrosis and corresponding statistics in *Pantoea ananatis*.

Gene	Annotation	Naive_p*	Bonferroni _p*	Benjamini _H_p*
*hvaA*	Hypothetical	2.13E-14	2.41E-10	2.68E-11
*pepM*	Phosphoenolpyruvate mutase	4.53E-13	5.12E-09	1.46E-10
*pavC*	Flavin-dependent monooxygenase	2.13E-14	2.41E-10	2.68E-11
*pavD*	Phosphonomethyl malate synthase	2.13E-14	2.41E-10	2.68E-11
*pavE*	3-isopropylmalate dehydratase large subunit	4.53E-13	5.12E-09	1.46E-10
*pavF*	3-isopropylmalate dehydratase small subunit	2.13E-14	2.41E-10	2.68E-11
*pavG*	SAM dependent methyltransferase	2.58E-15	2.91E-11	9.70E-12
*pavH*	GNAT family N-acetyltransferase	1.57E-13	1.78E-09	1.37E-10
*pavI*	ATP-grasp domain containing protein	2.13E-14	2.41E-10	2.68E-11
*pavJ*	MFS transporter	2.13E-14	2.41E-10	2.68E-11
*pavK*	Hypothetical	2.13E-14	2.41E-10	2.68E-11
*pavL*	Flavin reductase	1.57E-13	1.78E-09	1.37E-10
*pavM*	ATP-grasp domain containing protein	5.79E-14	6.54E-10	6.54E-11
*pavN*	Transposases	1.57E-13	1.78E-09	1.37E-10

*p-Value.

### Annotation

Prokka cannot distinguish between complete and truncated genes during annotation, which may potentially result in some genes to be wrongly annotated. Hence, once the pan-genome was defined, we carried out a blast-based annotations of sequences that constituted the core and accessory genomes. We annotated core, soft-core, shell and cloud genes. A total of 2,705 core, 3,293 soft-core, 2,058 shell and 3,503 cloud sequences were annotated successfully, mapped and assigned at least one gene ontology (GO) id and GO slim category (Supplementary Tables 14 - 17). GO analyses of the top terms revealed that metabolic process represented the most abundant category, followed by cellular processes under BP (Supplementary Figure 8). Under BP, the cellular amino acid metabolic process was specific to core genes and not present in soft-core, shell and cloud genes (Supplementary Figure 8). For genes to which MFs could be assigned, catalytic activity was the most abundant category, followed by binding. Transmembrane transporter and oxidoreductase activity; however, was not observed in the shell genes. GO analyses showed that cellular anatomical entities were represented the most abundantly under the CC category (Supplementary Figure 8).

### High Virulence/PASVIL and *alt* Genes Presence and Absence

**FIGURE 5:**
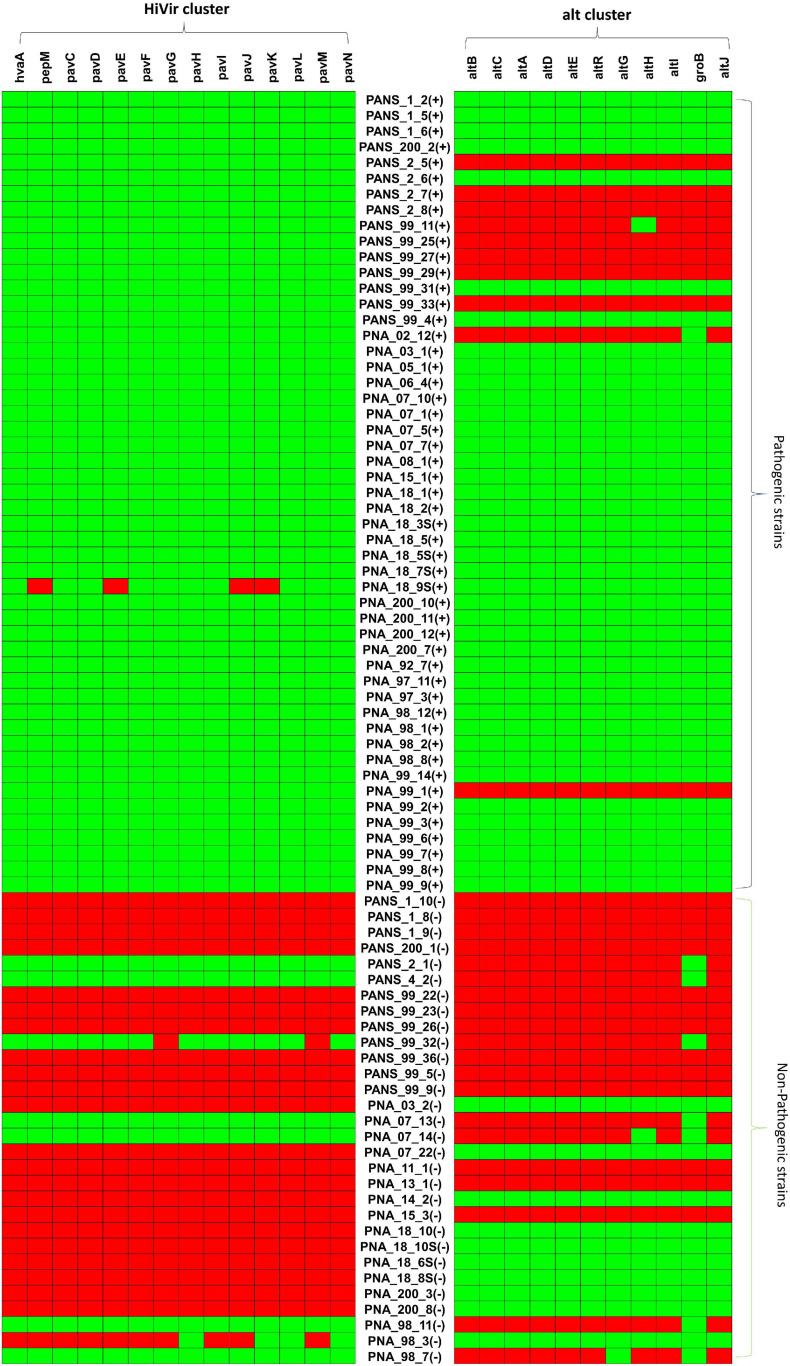
Pictorial representation of presence and absence of HiVir/PASVIL genes associated with red onion scale necrosis and *alt* genes associated with colonization in necrotic zone in pathogenic- and non-pathogenic strains of *Pantoea ananatis*. Presence and absence matrices of genes in the two gene clusters are aligned with each *P. ananatis* strain, ‘+’ represent the pathogenic and ‘–‘ represent the non-pathogenic strains.

Overall, 80.3% (41 of 51) of the pathogenic strains had both HiVir/PASVIL (*n* = 14 genes) and *alt* (*n* = 11 genes) clusters. However, none of the non-pathogenic strains had both of these gene clusters. Alternatively, none of the pathogenic strains showed the absence of both gene clusters (Figure 5).

The HiVir/PASVIL cluster was conserved in 98% (50 of 51) of the pathogenic strains. The pathogenic strain (PNA_18_9s) showed a partial loss of HiVir/PASVIL genes (*pepM, pavE, pavJ* and *pavK* genes). Absence of these genes could either be due to assembly artifacts or attributed to inconsistent and weak phenotypes (negligible red-scale clearing). Among the 30 non-pathogenic strains, 73.3% (*n* = 22) lacked a complete HiVir/PASVIL cluster and 6.6% (*n* = 2) of the strains showed the presence of only a subset (one or more) of the genes in the HiVir/PASVIL cluster. Interestingly, 20% (*n* = 6) of the non-pathogenic strains possessed a conserved complete HiVir/PASVIL cluster (Figure 5).

Among the pathogenic strains, the *alt* cluster was conserved in 80.3% (*n* = 41), absent in 15.6% (*n* = 8) strains and partially present in two strains (with just one gene present). However, among the non-pathogenic strains, the *alt* cluster was present in 33.3% (*n* = 10), absent in 43.3% (*n* = 13) and partially present (one or two out of 11 *alt* genes present) in 23.3% (*n* = 7) of the strains. Since only one or two genes in the *alt* gene cluster were present in the seven strains (PANS_2_1, PANS_4_2, PANS_99_32, PNA_7_13, PNA_7_14, PNA_98_11, PNA_98_7), these strains were considered as negative for the presence of the conserved *alt* cluster (Figure 5). As a result, 66.6% (*n* = 20) of the non-pathogenic strains lacked the *alt* cluster.

## Discussion

### Pan-Genome of *P. ananatis*

Building a pan-genome is important as it tremendously aids in gene discovery and helps in understanding the genome architecture of a species. A pan-genome represents the genomic repertoire of a species that can address questions related to varied phenotypes exhibited by individuals of that species. Current pan-genome study of *P. ananatis* that causes center rot in onions has helped in defining a conserved core genome and a dynamic accessory genome represented by PAVs. Pan-genome analysis of 81 genomes (*n* = 51 pathogenic, *n* = 30 non-pathogenic) revealed that the core genome was stabilized with 3,153 gene clusters while the pan-genome expanded continuously as a result of the addition of gene clusters indicating an open-pangenome. Earlier pan-genome studies identified similar but a little higher number of core genes while using a much smaller number of *P. ananatis* genomes (De Maayer et al., 2014; Sheibani-Tezerji et al., 2015; Stice et al., 2018). The ANI of ≥ 95% is a benchmark to classify organisms of the same species (Richter and Rosselló-Móra, 2009; Sangal et al., 2016), whereas, genomes of organisms with ANI values < 94% suggests that the organisms belong to different species (Sangal et al., 2016). In this study, ANI ranged from 99.0-99.9% and AAI ranged from 96-99%, which not only suggested low core genome diversity among the 81 *P. ananatis* strains but also confirmed that the strains belonged to *P. ananatis* species.

### Phylogenetic Analysis and Identification of HGTs, Genomic Islands, Pathogenicity and Symbiotic Factors in *P. ananatis*

Earlier, MLSA and rep-PCR assays showed limited genetic diversity, despite high phenotypic variation, among 50 *P. ananatis* strains (Stice et al., 2018). In the same study, the authors demonstrated that PAVs from the pan-genome analysis of 10 *P. ananatis* strains separated the pathogenic strains from the non-pathogenic strains, which was not observed when the core genome was used. Phylogenetic studies conducted so far in *P. ananatis* have relied only on PAVs, which are mainly horizontally acquired (Welch et al., 2002) contrary to the vertically inherited SNPs (Straub et al., 2021). In the current study, we therefore conducted a comparative phylogenetic study using core genome SNPs, PAVs and wgMLST approach. Core SNPs are relied to infer phylogeny as they represent the vertically inherited portion of the genome in the population (Didelot and Wilson, 2015; Straub et al., 2021). It was however observed that pathogenic strains clustered together with the non-pathogenic strains (in some groups in the phylogenetic tree) based on core SNPs. We expected core genome SNP variations to distinguish the pathogenic and non-pathogenic strains based on their vertically inherited evolutionary history. It could be argued that presence of homologous recombination and HGT events in the core genome could have distorted the phylogenetic relationship thus resulting in an unexpected phylogenetic classification of pathogenic and non-pathogenic strains. However, the possibility of HGT based distortion can be ruled out because our core SNP analysis was not based on a single reference genome. Use of a reference genome-based SNP calling approach can result in SNPs that are present in core genome of some strains that have horizontally acquired genes similar to reference genome but not present in other strains that do not have such horizontally acquired genes (Straub et al., 2021). In the current study, the core genome region of 81 strains was aligned (non-reference based) to identify the variant and invariant sites in all the strains. Pathogenicity phenotype in *P. ananatis* is therefore concluded to be a result of PAVs of pathogenicity-related genes in strains, which may be a result of various HGT events during bacterial evolution more than the vertically inherited SNPs.

Whole genome multi locus sequence typing (wgMLST) (Maiden et al., 2013) is an extended concept of the traditional MLST (Aanensen and Spratt, 2005) and is considered as an ideal approach to sort out WGS data and generate genetic layouts that are portable and comparable among laboratories (Liu et al., 2016). Using wgMLST, pathogenic and non-pathogenic *P. ananatis* strains were resolved. The wgMLST approach is based on the presence and absence of an allele. This approach in a way is complementary to phylogenomic analysis based on PAVs. Therefore, it further supports our findings that PAVs alone could provide a better phylogenomic resolution of pathogenic and non-pathogenic strains than SNPs.

Unrelated bacteria sharing a common environment are known to engage in frequent HGT (Smillie et al., 2011). HGT and gene loss are the key processes in bacterial evolution. HGT primarily occurs through lateral gene transfer, which drives both phenotyping changes and subsequent adaptation when the acquired genes confer new traits resulting in diversification of that lineage into a new environment (Treangen and Rocha, 2011; Nowell et al., 2014). The dynamics of genome fluctuation in *P. ananatis* can be attributed to HGT. Further, existence of pathogenic non-onion host strains (designated as PANS) could be the result of acquired pathogenicity from the pathogenic *P. ananatis* through HGT. HGT can increase the genetic variability if the donor is dispersed from a foreign population or is distantly related, conversely, HGT can homogenize a population in terms of gene content if it spreads the genetic material throughout the population (Van Rossum et al., 2020). The strain, PNA_98_12 in our study possessed maximum branch length (PAV based phylogeny) and also resulted in highest number of HGT events suggesting that the strains might possess novel gene content, which is distributed to the population in order to homogenize the population. Other strains with similar longer branch lengths, PNA_98_11, PNA_98_1, PANS_99_27 and PNA_18_10 could also possibly be involved in similar phenomenon of homogenizing the population based on lateral transfer of genes via HGT implying soft selective sweeps (multiple beneficial alleles at a locus gain prevalence, replacing standing genetic variation in the population). However, further studies are required to confirm the occurrence in *P. ananatis*.

Genomic islands, also known as clusters of bacterial or archaeal genes are of probable horizontal origin and are of interest as they contain the genes for virulence (pathogenicity), symbiosis and metabolism (Bertelli et al., 2019). We could predict the genomic islands, and pathogenicity and symbiotic factors for two *P. ananatis* strains (PNA_99_3 and PNA_99_14) that were involved in the maximum number of HGT events. We found that some of the pathogenic genes that were identified to play a significant role in causing red-scale necrosis of onions using pan-GWAS were also the part of the predicted genomic islands and pathogenicity factors.

### Pan-GWAS of *P. ananatis*

Bacterial phenotypes, in general, can be linked to the presence or absence of genes that are inherited through either descent or lateral gene transfer (Tettelin et al., 2005). We conducted pan-GWAS analysis to predict and associate genes related to pathogenicity in onion. Pan-genome PAVs were utilized to associate onion pathogenicity phenotypes (determined using a red-onion scale necrosis assay). The 14 strongly associated genes that were identified as a part of the HiVir/PASVIL cluster were annotated as *hvaA*, *pepM*, *pavC-N* (Takikawa and Kubota, 2018). These associated genes coded for phosphonate metabolism, metabolism of plant-derived aromatic compounds, monooxygenases, a methyltransferase, leucine biosynthesis and an L-amino acid ligase. Association of HiVir/PASVIL genes using this pan-genome *in silico* approach corroborated earlier findings of the roles of these genes in *P. ananatis*-pathogenicity in onion (Asselin et al., 2018; Polidore et al., 2021). Rest of the 28 associated genes were regarded as ‘novel’ as these were not identified in any of the previous pan-genome investigations. The eight annotated genes (part of 28 novel genes) code for: murein tetrapeptide carboxypeptidase, which is involved in the peptidoglycan recycling pathway (Perna et al., 2001); pyridoxal-4-dehydrogenase (*pld_1*) that belongs to aldo/keto reductase family and is involved in synthesis of 4-pyridoxate from pyridoxal (Yokochi et al., 2004); chromosome partitioning ATPase (coded by *soj3*) involved in the genome maintenance (Charaka and Misra, 2012); XerD protein is a site-specific tyrosine recombinase involved in cell division (Cerro et al., 2013); N-acetylmuramoyl-L-alanine amidase (coded by *amiD*) that belongs to glycosyl hydrolase family and is involved in cell wall macromolecule and peptidoglycan catabolic process (Wilkes et al., 2010); conjugal transfer protein (coded by *traR*) with zinc ion binding ability (Chen et al., 2019); the tibA adhesin, which induces bacterial aggregation and biofilm formation (Sherlock et al., 2005); and LysR transcriptional regulatory family protein involved in DNA-binding transcription factor activity (coded by *dmlR*) (Moura et al., 2017). Further functional analysis is warranted to understand their roles in onion pathogenicity.

### Comparative Genomics of HiVir/PASVIL Cluster and Role of Individual Genes in the Cluster

Comparative genomic analysis showed a trend in the presence or absence of complete/conserved HiVir/PASVIL cluster genes in pathogenic vs. non-pathogenic *P. ananatis* strains. Interestingly, 20% (*n* = 6) of the non-pathogenic strains possessed a conserved complete HiVir/PASVIL cluster. If the presence of a complete cluster is correlated with onion pathogenicity, then it is difficult to explain the presence of a complete cluster of genes in these non-pathogenic strains. It is possible that the HiVir/PASVIL genes in the cluster in non-pathogenic strains are not expressed or are non-functional, which may require further investigation and confirmation.

Phosphoenolpyruvate mutase (*pepM*) is involved in phosphonate biosynthesis. Organophosphonates are synthesized as secondary metabolites in certain prokaryotes to function as antibiotics, and can have specialized roles in pathogenesis or signaling (Hilderbrand, 1983). Phosphonate metabolites are derived from phosphonopyruvate, which in turn is formed from phosphoenolpyruvate (PEP) by the action of PEP mutase (PepM). Asselin et al. (Asselin et al., 2018) identified a *pepM* gene as the first pathogenicity factor associated with the fitness of *P. ananatis* as well as with symptom development in infected onion leaves and bulbs. Deletion of *pepM* or inactivation of *pavJ* gene resulted in loss of the ability to cause lesions on onion foliage and bulbs. Furthermore, growth of the deletion mutant in onion leaves was significantly reduced compared with the wild-type *P. ananatis* strain. This pan-genome *in silico* study corroborated the association of *pepM* gene with onion pathogenicity, using a diverse panel of *P. ananatis* strains. The *pepM* gene was present in 50 of 51 pathogenic strains, with the exception of a strain PNA 18-9 s. This strain also lacked *pavE*, *pavJ*, and *pavK*. If it is not an assembly artifact, the absence of *pepM* along with four other genes in the HiVir/PASVIL cluster in this strain could be the reason for a compromised red scale necrosis phenotype (weak pathogenicity). This observation also indicated the presence of a potential alternative pathogenicity factor than *pepM*, which requires further investigation. For the non-pathogenic strains of *P. ananatis*, *pepM* gene was absent in 23 of 30 strains. Despite the presence of *pepM* gene and, in some cases, the entire HiVir/PASVIL cluster (six of 30 strains), these strains displayed a non-pathogenic phenotype. These observations suggest that these genes may be non-functional in these strains, which warrants further research.

A monooxygenase and a flavin reductase enzyme belonging to the two-component non-hemeflavin-diffusible monooxygenase (TC-FDM) family were found to be associated with onion pathogenicity using pan-GWAS study. The monooxygenase and the reductase associated are nitrilotriacetate monooxygenase coded by *nta*A (similar to *pavC* in the HiVir/PASVIL cluster of *P. ananatis*) and flavin reductase, a flavin:NADH oxidoreductase component of 4-hydroxyphenylacetate (4-HPA) 3-monooxygenase coded by *hpa*C (similar to *pavL* in the HiVir/PASVIL cluster of *P. ananatis*). Nitrilotriacetate monooxygenase was reported previously in the genomic region referred as WHOP (woody host of *Pseudomonas* spp.) in a *Pseudomonas syringae* complex (Caballo-Ponce et al., 2017b). This region is associated with strains of *P. syringae* that infect woody host plants, and is absent in strains infecting herbaceous tissues. This gene, along with other genes present in the WHOP region, is responsible for the fitness and virulence of *Pseudomomas savastanoi* pv. *savastanoi* in woody olive trees, but not in non-woody olive trees (Caballo-Ponce et al., 2017a; Caballo-Ponce et al., 2017b). Nitrilotriacetate monooxygenase is known to catabolize plant-derived aromatic compounds and help bacteria to adapt to woody host tissues (Ramos et al., 2012). On the contrary, *P. ananatis* colonize foliar and bulb tissue in onion, which are non-woody, therefore, it was intriguing to find this gene associated with pathogenicity in onion, an herbaceous plant.

The HiVir/PASVIL gene *pavG* in *P. ananatis* has an annotated function for a class-I S-adenosyl-L-methionine (SAM)-dependent methyltransferase. We presume that *pavG* is responsible for the esterification of phosphonates synthesized in *P. ananatis*, led by *pepM*, based on the fact that the di-anionic form of phosphonates interferes with the metabolic intermediates and carboxylates of antibacterial compounds (Metcalf and van der Donk, 2009; Lee et al., 2010; Yu et al., 2013). To counteract this problem, microbes either synthesize phosphinites (with a double bond between the C and P instead of a single bond) or carry out esterification of phosphonates. Phosphonate esterification appears to be an obvious mechanism operational in *P. ananatis* because of the presence of *pavG* in the HiVir/PASVIL cluster. SAM dependent O-methyltransferase has been shown to methylate a variety of phosphonates (1-hydroxyethylphosphonate, 1, 2-dihydroxyethylphosphonate, and acetyl-1- aminoethylphosphonate) (Lee et al., 2010). Therefore, there is a high possibility of involvement of SAM methyltransferase in methylation of the phosphonate produced in *P. ananatis*. Further studies are required to characterize the type of phosphonate and its methylation in order to understand the mechanism of SAM methyltransferase and *pepM* in causing red-scale necrosis. Another role that *pavG* could be playing is methylation of other HiVir/PASVIL genes that renders them inactive despite their presence in the cluster. We hypothesize that the inactivity of HiVir/PASVIL genes may be due to methylation of genes carried out by SAM dependent methyltransferase in non-pathogenic strains of *P. ananatis*, implying a secondary role of *pavG* in strains non-pathogenic to onion. Methylation profiling will help evaluate this hypothesis.

The HiVir/PASVIL gene *pavI* is similar to RizA an L-amino-acid ligase (LAL) from *Bacillus subtilis* that participates in the biosynthesis of rhizocticin, a phosphonate oligopeptide antibiotic and possess L-arginyl-L-2-amino-5-phosphono-3-cis-pentenoic acid (Kino et al., 2009). Although, the functional role of *pavI* is yet to be characterized in *P. ananatis*, it may play a role in the formation of anti-microbial secondary metabolites of “phosphonate derivatives.” LAL is a member of the ATP-dependent carboxylate–amine/thiol ligase superfamily (Galperin and Koonin, 1997), and catalyzes the ligation reaction, which involves an aminoacyl-phosphate intermediate, in an ATP-dependent manner (Fan et al., 1995). LALs contain the ATP-grasp fold, which is composed of three conserved domains referred to as the A-domain (N-terminal domain), the B-domain (central domain) and the C-domain (C-terminal domain). These three domains commonly grasp the ATP molecule, and also provide binding sites for the Mg2 + ion and the amino-acid substrate (Kagawa et al., 2015).

The pan-GWAS approach used in this study did not associate the genes in *alt* cluster with the onion pathogenic phenotype using the red-scale necrosis assay. This may be because of the type of phenotyping assay utilized in this study. The red-scale necrotic assay has been shown to be induced by the HiVir/PASVIL cluster (Stice et al., 2020). However, the *alt* cluster comes into play after the onset of necrosis, when endogenous antimicrobial sulfur compounds are produced by damaged onion cells. In this scenario, the *alt* cluster helps *P. ananatis* to survive and colonize onion tissue. *Pantoea ananatis* uses 11 *alt* cluster genes associated with the sulfur metabolism that impart tolerance to the thiosulfinate ‘allicin’ produced by damaged onion cells (Stice et al., 2020). The presence of the *alt* cluster in 80% (*n* = 41) of the onion pathogenic strains, and its absence in 67% (*n* = 20) of the non-pathogenic strains, suggests a potential involvement in bacterial virulence. However, the *alt* cluster alone is not sufficient for the onion pathogenic phenotype, as 33% (*n* = 10) of the non-pathogenic strains did not exhibit any evidence of pathogenicity in the onion red scale assay despite possessing a completely conserved *alt* cluster. These ten strains; however, did not contain complete or partial HiVir cluster except for one strain (PNA_98_3), which possessed a partial HiVir cluster.

## Conclusion

In this study, we used the pan-GWAS approach to predict genes associated with onion-pathogenicity in *P. ananatis*. We concluded that the HiVir/PASVIL genes are associated with onion-pathogenicity as determined by the red-scale necrosis assay, and the *alt* gene cluster alone is not sufficient for pathogenic phenotype. Also, HiVir/PASVIL gene expression is potentially regulated, and the mere presence of the HiVir/PASVIL cluster does not guarantee a strain to be pathogenic on onion. In addition, a large repertoire of accessory genes identified in these strains may aid *P. ananatis* in diverse niche-adaptation and potentially in host-range expansion. The pan-GWAS pipeline can be deployed to characterize *P. ananatis* strains pathogenic to other plant hosts. We observed HGT events as major contributing factor for PAVs resulting in diversification of *P. ananatis* strains. Further integration of ‘omics’ technologies will provide deeper insights into the identification of novel pathogenicity and virulence factors in *P. ananatis* populations that cause center rot of onion. Whole transcriptome and proteome studies are required to understand the expression and function of identified pathogenicity and virulence factors in *P. ananatis*. A time-course based transcriptomic studies will reveal the temporal expression of pathogenicity and virulence genes as infection progresses. Proteomic studies will be used to validate transcriptomic expression of these genes and identify gene products.

## Data Availability Statement

The datasets presented in this study can be found in online repositories. The names of the repository/repositories and accession number(s) can be found in the article/Supplementary Material.

## Author Contributions

GA and BD conceived the project. GA performed the bioinformatics analyses and complied the manuscript. DC maintained the bacterial cultures, isolation of strains, and phenotyping of the 81 strains. SS and BK contributed in planning, designing the experiment, and manuscript revision. BM and SV contributed to the discussion. GA, RG, and BD designed and finalized the manuscript. BD planned the project, secured extramural funds, and revised and submitted manuscript. All authors contributed to the article and approved the submitted version.

## Conflict of Interest

The authors declare that the research was conducted in the absence of any commercial or financial relationships that could be construed as a potential conflict of interest.

## Publisher’s Note

All claims expressed in this article are solely those of the authors and do not necessarily represent those of their affiliated organizations, or those of the publisher, the editors and the reviewers. Any product that may be evaluated in this article, or claim that may be made by its manufacturer, is not guaranteed or endorsed by the publisher.

## Footnotes

1
https://github.com/songweizhi/BioSAK/tree/master/BioSAK_tutorial/Demo_tree_visualization_with_iTol

## Supplementary Material

Supplementary Figure 1 Onion red scale necrosis shown by pathogenic strains of *P. ananatis*. Three replicates (rep 1, rep 3, and rep 3) are shown for representative pathogenic stains (PNA 99-14, PNA 99-2, PNA 97-11, 99-1, and PANS 200-1) used to inoculate the red onion scales. Pathogenic strains showed scale clearing (bleaching out phenotype) whereas non-pathogenic strain did not show any symptoms. The positive control (PNA 97-1) and negative control (sterile water) is represented by “+” and “−”, respectively.

Supplementary Figure 2 Raw-read data generated, and filtered reads retained after stringent quality filtering using Trimmomatic.

Supplementary Figure 3 Pan-genome analysis of 81 *Pantoea ananatis* genomes. **(A)** Genes contributed to pan-genome by individual genomes. **(B)** Distribution of gene (cluster) sizes as a function of the number of genomes they contain showing the partition of OMCL pan-genomic matrix into shell, cloud, soft-core and core compartments.

Supplementary Figure 4 Average nucleotide identities (ANI) of the coding DNA sequences and average amino acid identities (AAI) of the protein coding genes of 81 *Pantoea ananatis*. **(A)** Heatmap was generated using the identity matrix calculated by get_homologues.pl using the BLAST scores representing the degree of similarity of the genomes based on ANI. **(B)** Heatmap showing the degree of similarity of the genomes based on the AAI calculated using BLASTP scores implemented in get_homologues.pl. Heatmaps were derived from the ANI and AAI matrix based on the pan-genome matrices. High similarity is represented by lighter color (light yellow to white) and low similarity is represented by dark orange to light orange shade. Vertical and horizontal axes are labeled with names of 81 strains of *P. ananatis*.

Supplementary Figure 5 Dendrogram of 51 red-onion scale necrotic and 30 red-onion scale non-necrotic strains of *P. ananatis* based on shell genes. Strains highlighted in green are non-pathogenic and ones in red are pathogenic.

Supplementary Figure 6 Dendrogram of 51 red-onion scale necrotic and 30 red-onion scale non-necrotic strains of *P. ananatis* based on cloud genes. Strains highlighted in green are non-pathogenic and ones in red are pathogenic.

Supplementary Figure 7 Comparative phylogeny of 81 pathogenic and non-pathogenic strains of *Pantoea ananatis* based on core genome SNPs and presence and absence variations. **(A)** Phylogenetic tree constructed using core SNPs using RAxML. **(B)** Phylogenetic tree constructed using PAVs using RAxML. Numerical values in decimal represent the branch length. Longer branch length mean higher genetic divergence. ‘P’ represents pathogenic and ‘NP’ represents non-pathogenic strains. Strains labeled as PANS represent their non-onion origin. The rest were identified from onions (PNA).

Supplementary Figure 8 Top GO terms: A bar chart representing the GO terms according to the number of annotated sequences. Panel A-D shows the function of genes assigned to biological process; panel E-H shows the function of genes assigned to molecular function and panel I-L represent the function of genes assigned to cellular component.

Supplementary Table 1 Details of raw and filtered data generated for each sample of *P. ananatis* used in this study.

Supplementary Table 2 Total number of core and accessory genes shared by each strain of *P. ananatis* used in this study.

Supplementary Table 3 List of putative horizontal gene transfers, donor and recipient genomes and, gene transfers between two genomes.

Supplementary Table 4 List of HGTs involving HiVir/PASVIL/analogous TCA genes.

Supplementary Table 5 Genomic islands in PNA 99_3 predicted by SGI-HMM implemented by IslandViewer 4.

Supplementary Table 6 Genomic islands in PNA 99_3 predicted by IslandPath-DIMOB implemented by IslandViewer 4.

Supplementary Table 7 Pathogenicity factors (virulence factors) in PNA_99_3 as predicted by GIPSy.

Supplementary Table 8 Symbiotic factors in PNA_99_3 as predicted by GIPSy.

Supplementary Table 9 Genomic islands in PNA 99_14 as predicted by SGI-HMM implemented by IslandViewer 4.

Supplementary Table 10 Genomic islands in PNA 99_14 predicted by IslandPath-DIMOB implemented by IslandViewer 4.

Supplementary Table 11 Pathogenicity factors (virulence factors) in PNA_99_14 as predicted by GIPSy.

Supplementary Table 12 Symbiotic factors in PNA_99_14 as predicted by GIPSy.

Supplementary Table 13 List of 42 genes associated with red-onion scale necrosis caused by pathogenic strains of *P. ananatis* based on *P*-values.

Supplementary Table 14 Annotation and mapping of representative core genes.

Supplementary Table 15 Annotation and mapping of representative soft-core genes.

Supplementary Table 16 Annotation and mapping of representative shell genes.

Supplementary Table 17 Annotation and mapping of representative cloud genes.

Supplementary Table 18 Life Identification Number (LIN) Ids assigned to *P. ananatis* strains submitted to LIN base.
