# Supplementary figures and images for: Pan-Genome-Wide Analysis of Pantoea ananatis Identified Genes Linked to Pathogenicity in Onion
Source: Front Microbiol. 2021 Aug 19;12:684756. doi: 10.3389/fmicb.2021.684756 (PMC8417944; doi:10.3389/fmicb.2021.684756)

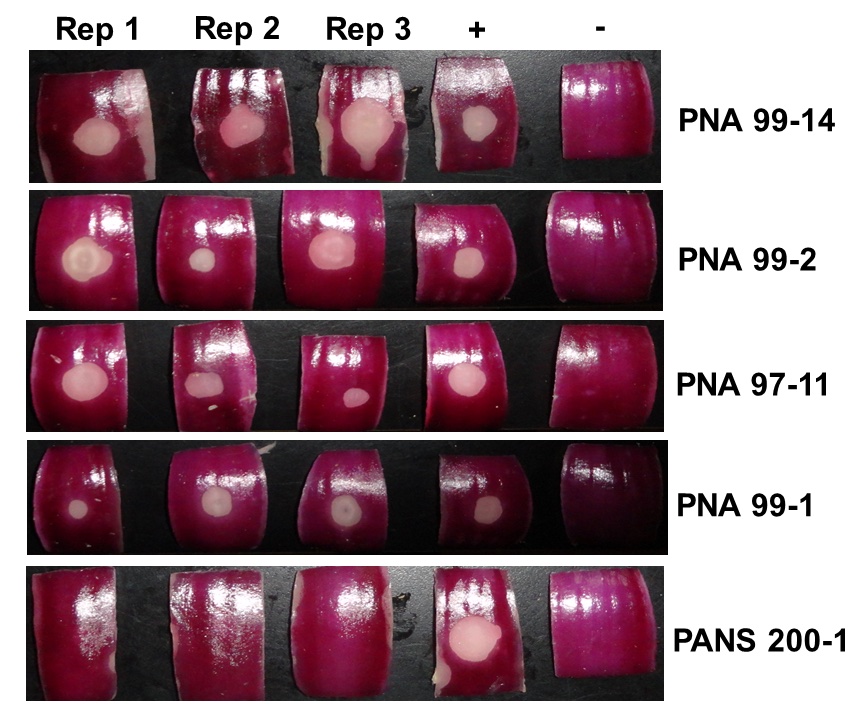

Supplement: Supplementary Figure 1 — Onion red scale necrosis shown by pathogenic strains of P. ananatis. Three replicates (rep 1, rep 3, and rep 3) are shown for representative pathogenic stains (PNA 99-14, PNA 99-2, PNA 97-11, 99-1, and PANS 200-1) used to inoculate the red onion scales. Pathogenic strains showed scale clearing (bleaching out phenotype) whereas non-pathogenic strain did not show any symptoms. The positive control (PNA 97-1) and negative control (sterile water) is represented by “+” and “−”, respectively. [file Image_1.JPEG]

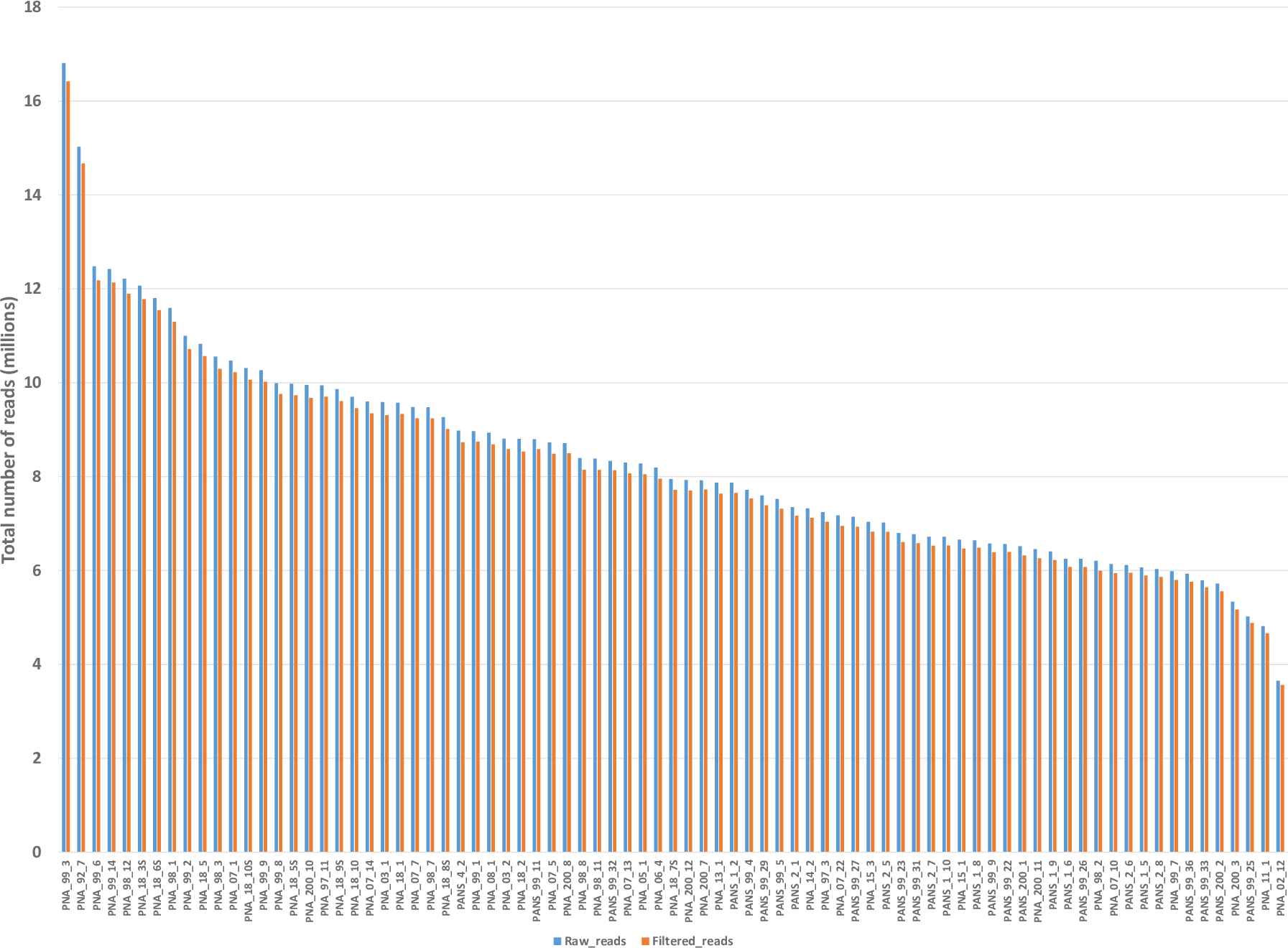

Supplement: Supplementary Figure 2 — Raw-read data generated, and filtered reads retained after stringent quality filtering using Trimmomatic. [file Image_2.TIF]

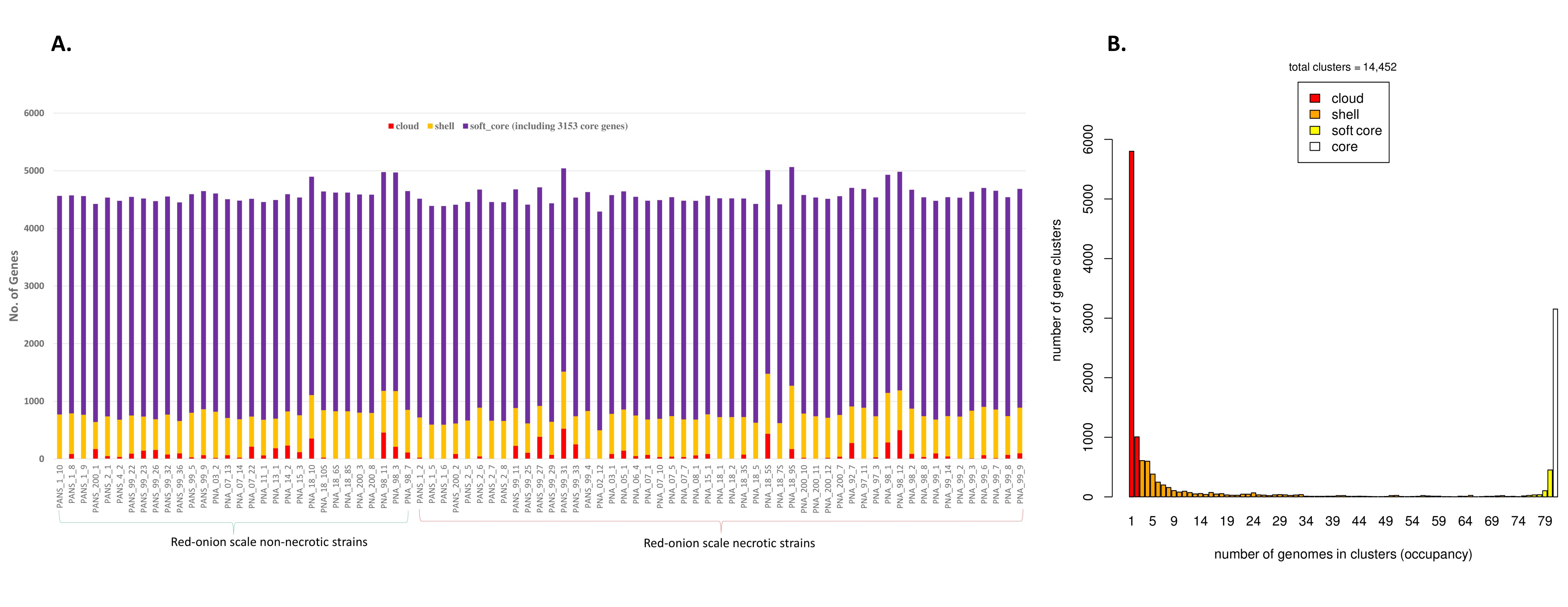

Supplement: Supplementary Figure 3 — Pan-genome analysis of 81 Pantoea ananatis genomes. (A) Genes contributed to pan-genome by individual genomes. (B) Distribution of gene (cluster) sizes as a function of the number of genomes they contain showing the partition of OMCL pan-genomic matrix into shell, cloud, soft-core and core compartments. [file Image_3.TIFF]

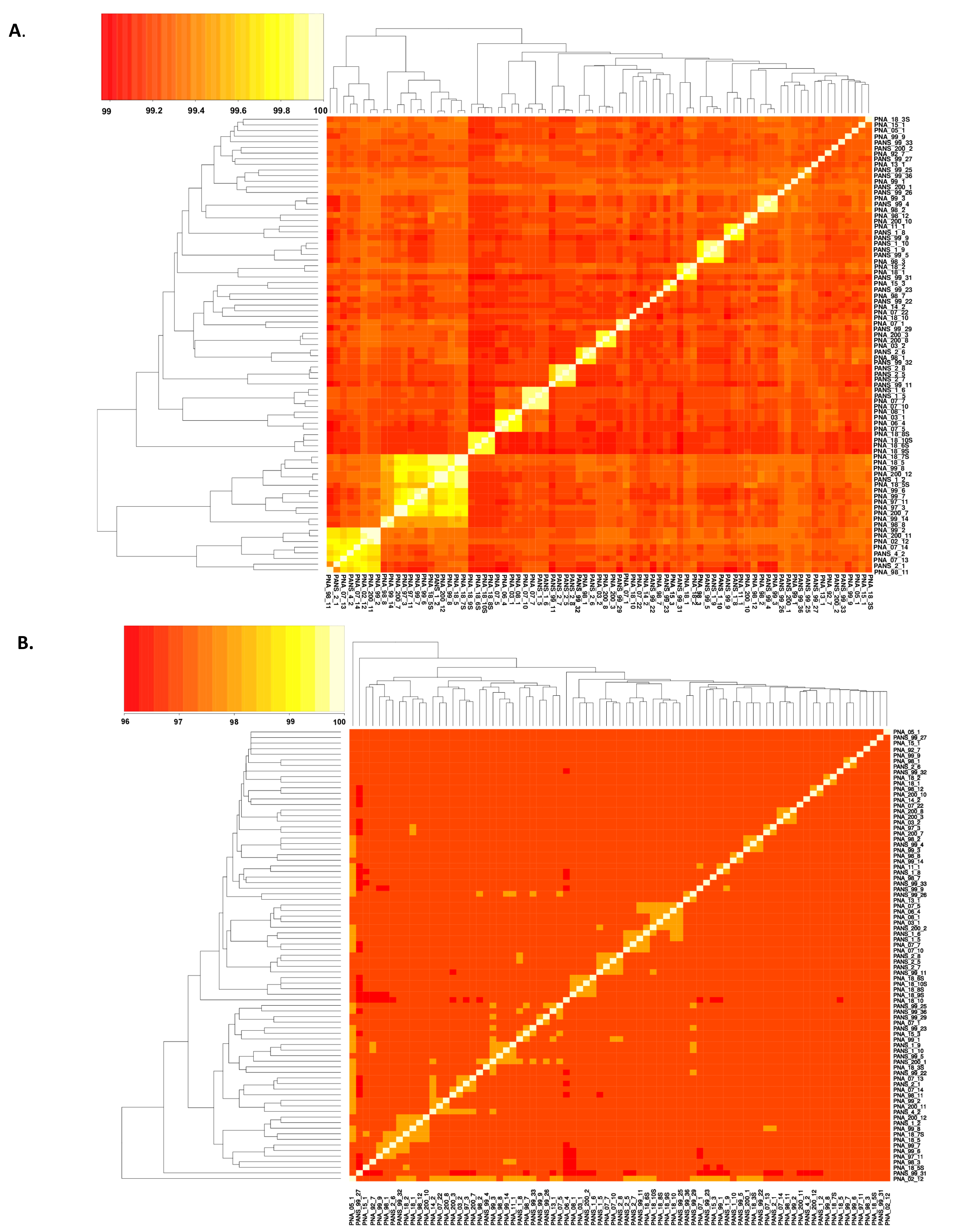

Supplement: Supplementary Figure 4 — Average nucleotide identities (ANI) of the coding DNA sequences and average amino acid identities (AAI) of the protein coding genes of 81 Pantoea ananatis. (A) Heatmap was generated using the identity matrix calculated by get_homologues.pl using the BLAST scores representing the degree of similarity of the genomes based on ANI. (B) Heatmap showing the degree of similarity of the genomes based on the AAI calculated using BLASTP scores implemented in get_homologues.pl. Heatmaps were derived from the ANI and AAI matrix based on the pan-genome matrices. High similarity is represented by lighter color (light yellow to white) and low similarity is represented by dark orange to light orange shade. Vertical and horizontal axes are labeled with names of 81 strains of P. ananatis. [file Image_4.TIFF]

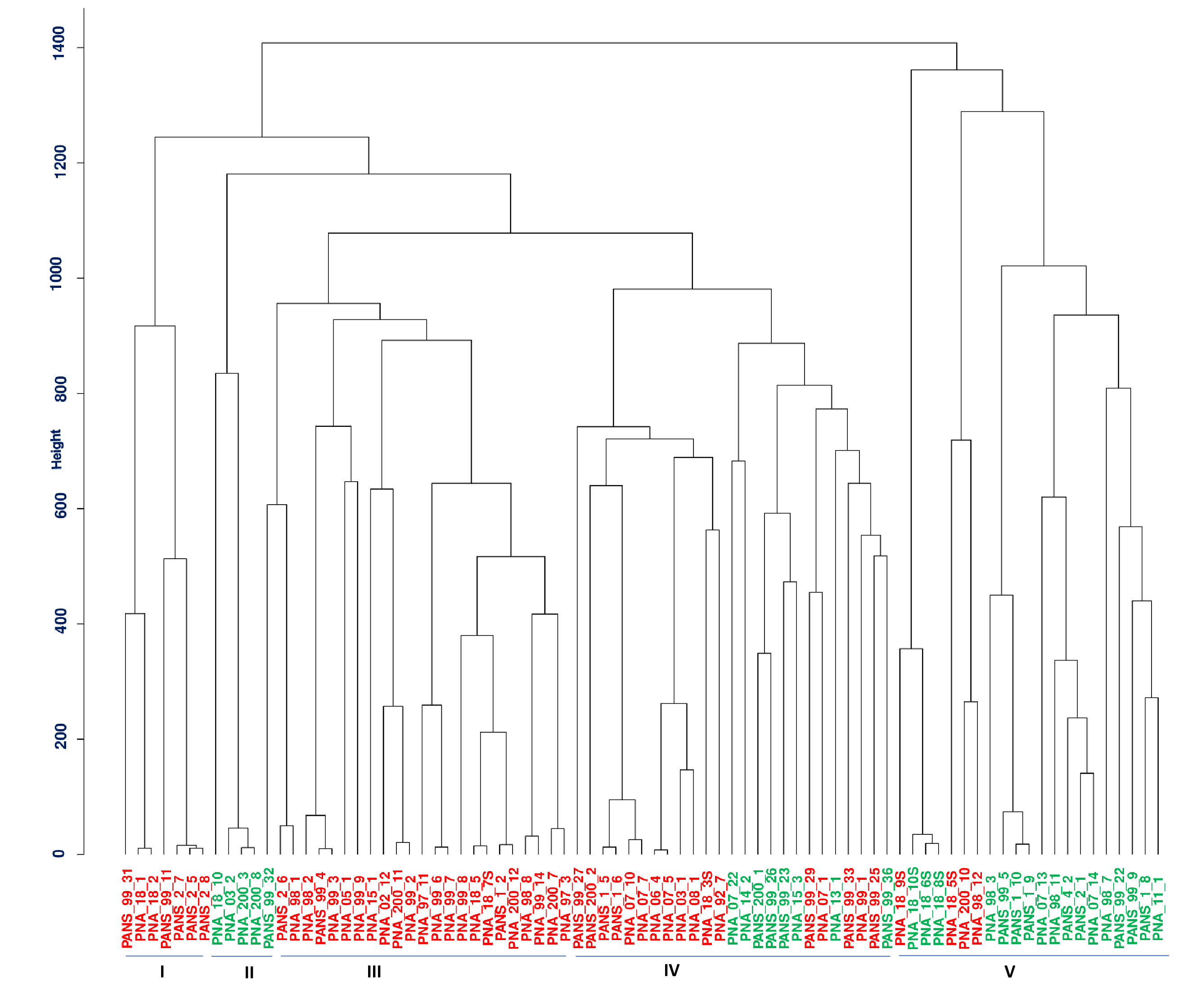

Supplement: Supplementary Figure 5 — Dendrogram of 51 red-onion scale necrotic and 30 red-onion scale non-necrotic strains of P. ananatis based on shell genes. Strains highlighted in green are non-pathogenic and ones in red are pathogenic. [file Image_5.TIFF]

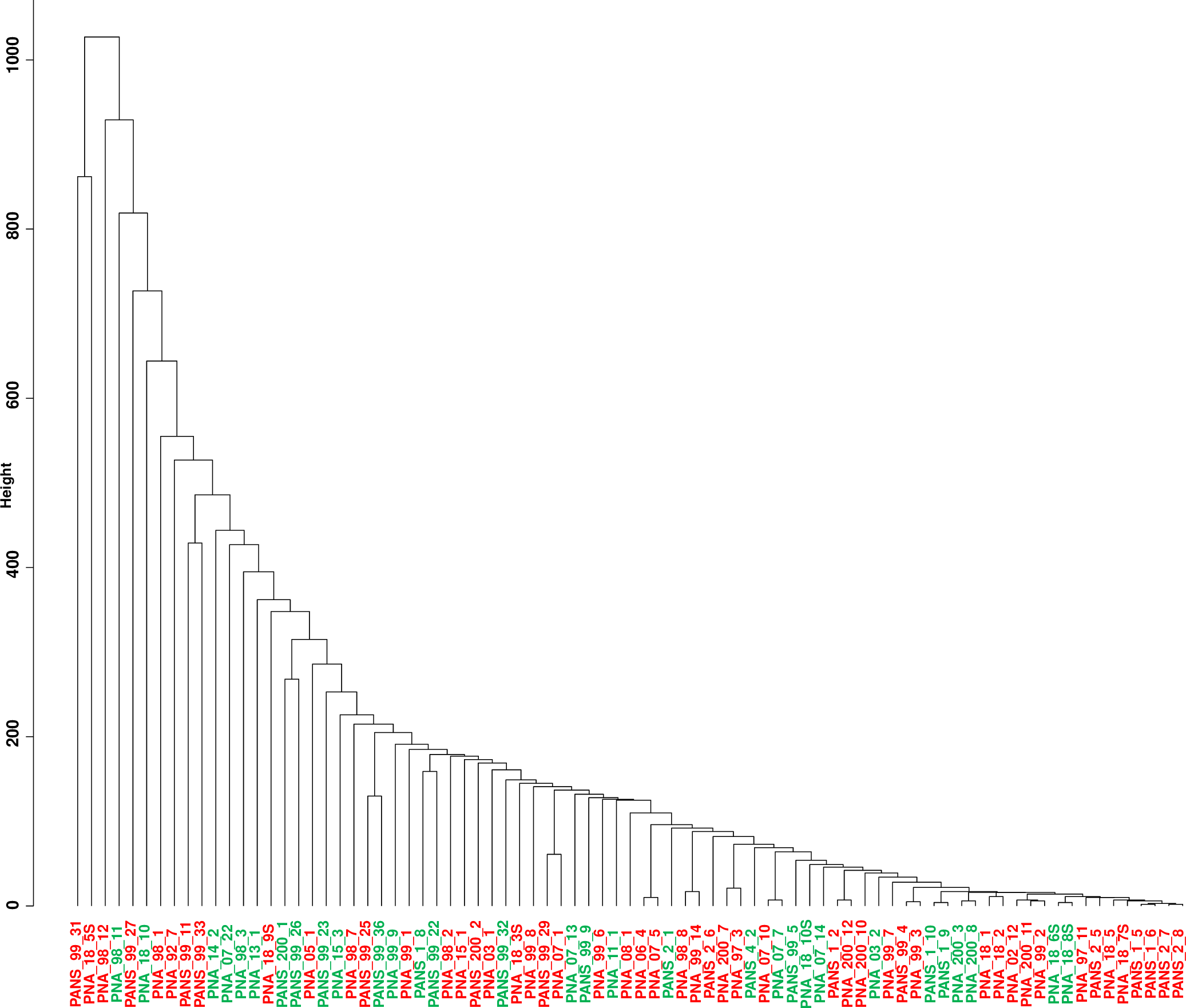

Supplement: Supplementary Figure 6 — Dendrogram of 51 red-onion scale necrotic and 30 red-onion scale non-necrotic strains of P. ananatis based on cloud genes. Strains highlighted in green are non-pathogenic and ones in red are pathogenic. [file Image_6.TIF]

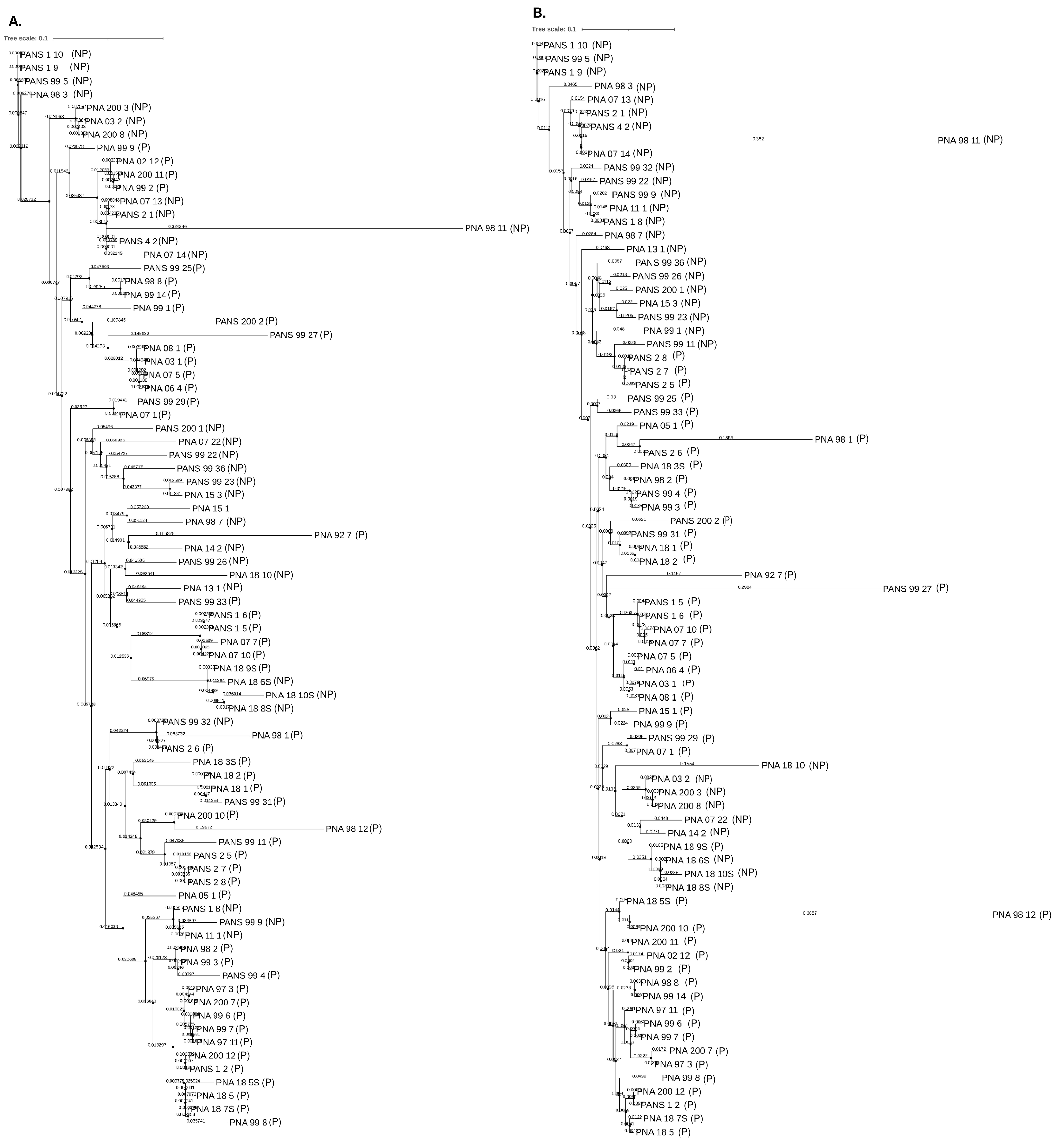

Supplement: Supplementary Figure 7 — Comparative phylogeny of 81 pathogenic and non-pathogenic strains of Pantoea ananatis based on core genome SNPs and presence and absence variations. (A) Phylogenetic tree constructed using core SNPs using RAxML. (B) Phylogenetic tree constructed using PAVs using RAxML. Numerical values in decimal represent the branch length. Longer branch length mean higher genetic divergence. ‘P’ represents pathogenic and ‘NP’ represents non-pathogenic strains. Strains labeled as PANS represent their non-onion origin. The rest were identified from onions (PNA). [file Image_7.TIFF]

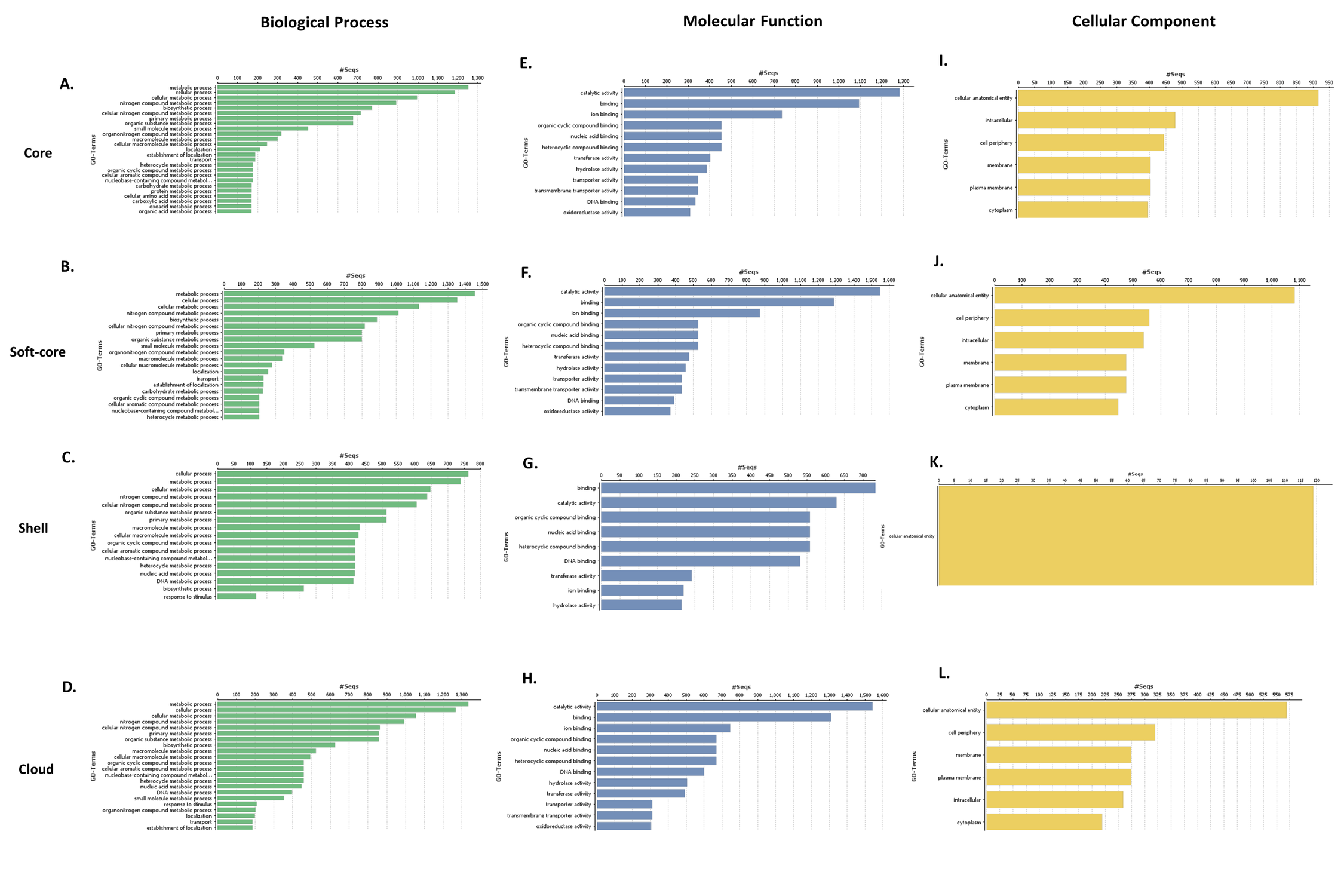

Supplement: Supplementary Figure 8 — Top GO terms: A bar chart representing the GO terms according to the number of annotated sequences. Panel A-D shows the function of genes assigned to biological process; panel E-H shows the function of genes assigned to molecular function and panel I-L represent the function of genes assigned to cellular component. [file Image_8.TIF]
